# Combination of Itacitinib or Parsaclisib with Pembrolizumab in Patients with Advanced Solid Tumors: A Phase I Study

**DOI:** 10.1158/2767-9764.CRC-22-0461

**Published:** 2023-12-19

**Authors:** Pamela Munster, Nicholas Iannotti, Daniel C. Cho, John M. Kirkwood, Liza C. Villaruz, Geoffrey T. Gibney, F. Stephen Hodi, Niharika B. Mettu, Mark Jones, Jill Bowman, Michael Smith, Mani Lakshminarayanan, Steven O'Day

**Affiliations:** 1 Department of Medicine, Division of Hematology/Oncology, UCSF, San Francisco, California.; 2 Hematology-Oncology Associates of Treasure Coast, Port St Lucie, Florida.; 3 NYU Laura & Isaac Perlmutter Cancer Center at NYU Langone, New York City, New York.; 4 UPMC Hillman Cancer Center Melanoma and Skin Cancer Program, Pittsburgh, Pennsylvania.; 5 UPMC Hillman Cancer Center, Pittsburgh, Pennsylvania.; 6 Georgetown Lombardi Comprehensive Cancer Center, Washington, District of Columbia.; 7 Dana-Farber Cancer Institute, Boston, Massachusetts.; 8 Duke Cancer Institute, Durham, North Carolina.; 9 Incyte Corporation, Wilmington, Delaware.; 10 John Wayne Cancer Institute of Providence, Saint John's Health Center, Santa Monica, California.

## Abstract

**Purpose:**

This phase Ib open-label, multicenter, platform study (NCT02646748) explored safety, tolerability, and preliminary activity of itacitinib (Janus kinase 1 inhibitor) or parsaclisib (phosphatidylinositol 3-kinase δ inhibitor) in combination with pembrolizumab [programmed death-1 (PD-1) inhibitor].

**Experimental Design:**

Patients with advanced or metastatic solid tumors with disease progression following all available therapies were enrolled and received itacitinib (Part 1 initially 300 mg once daily) or parsaclisib (Part 1 initially 10 mg once daily; Part 2 all patients 0.3 mg once daily) plus pembrolizumab (200 mg every 3 weeks).

**Results:**

A total of 159 patients were enrolled in the study and treated with itacitinib (Part 1, *n* = 49) or parsaclisib (Part 1, *n* = 83; Part 2, *n* = 27) plus pembrolizumab. The maximum tolerated/pharmacologically active doses were itacitinib 300 mg once daily and parsaclisib 30 mg once daily. Most common itacitinib treatment-related adverse events (TRAE) were fatigue, nausea, and anemia. Most common parsaclisib TRAEs were fatigue, nausea, diarrhea, and pyrexia in Part 1, and fatigue, maculopapular rash, diarrhea, nausea, and pruritus in Part 2. In patients receiving itacitinib plus pembrolizumab, four (8.2%) achieved a partial response (PR) in Part 1. Among patients receiving parsaclisib plus pembrolizumab, 5 (6.0%) achieved a complete response and 9 (10.8%) a PR in Part 1; 5 of 27 (18.5%) patients in Part 2 achieved a PR.

**Conclusions:**

Although combination of itacitinib or parsaclisib with pembrolizumab showed modest clinical activity in this study, the overall response rates observed did not support continued development in patients with solid tumors.

**Significance:**

PD-1 blockade combined with targeted therapies have demonstrated encouraging preclinical activity. In this phase I study, patients with advanced solid tumors treated with pembrolizumab (PD-1 inhibitor) and either itacitinib (JAK1 inhibitor) or parsaclisib (PI3Kδ inhibitor) experienced limited clinical activity beyond that expected with checkpoint inhibition alone and showed little effect on T-cell infiltration in the tumor. These results do not support continued development of these combinations.

## Introduction

Immune checkpoint receptors are expressed on tumor, stromal, and immune cells, and negatively regulate the immune response in the tumor microenvironment (TME; ref. 1). Immune modulation by targeting immune checkpoint receptors has demonstrated remarkable efficacy using single-agent or combination therapy in some cancer types, such as metastatic melanoma (2–4). Currently, multiple checkpoint inhibitor (CPI) antibodies have been approved by the FDA for treatment of patients with cancer, directed against CTL-associated antigen 4 (CTLA-4; ref. 5), programmed death-(ligand) 1 [PD-(L)1] (6–11), and lymphocyte-activation gene 3 (12). Overall, reported objective response rates (ORR) with anti-PD-(L)1 monotherapy range from 13% to 16% in head and neck squamous cell carcinoma (HNSCC), 20%–21% in urothelial carcinoma (UC), 19%–55% in non–small cell lung cancer (NSCLC), and 21%–40% in melanoma (2). Resistance to CPI monotherapy may be attributable to multiple immune inhibitory mechanisms operating concurrently within the TME (2). Therefore, combinations with CPIs may address an unmet need for improved therapeutic outcomes in patients with hematologic and solid tumors.

Excessive Janus kinase-signal transducer and activator of transcription (JAK/STAT) signaling within the TME may reduce antitumor responses, suggesting JAK enzymes could be potential therapeutic targets (13, 14). JAK1 inhibition with itacitinib (INCB039110) demonstrated reduced tumor volume in the immunocompetent, syngeneic PAN02 pancreatic cancer murine model (15). Furthermore, itacitinib enhanced antitumor response to anti-PD-L1 in this model, compared with single-agent itacitinib or anti-PD-L1 (15). Therefore, we tested the combined targeted inhibition of the PD-(L)1 pathway and JAK1 signaling.

PI3Kδ is enriched in hematopoietic cells, including lymphocytes and myeloid cells (16). Either genetic ablation or pharmacologic inhibition of PI3Kδ reduced tumor formation in multiple murine solid tumor models (B16 melanoma, Lewis lung carcinoma, EL4 thymoma, 4T1 breast cancer), with reported immune modulatory effects attributed to diminished activity of immunosuppressive regulatory T cells (Treg; ref. 17). PI3Kδ inhibition with parsaclisib (INCB050465) reduced tumor volume in the syngeneic PAN02 pancreatic tumor (15, 18), CT26 colorectal carcinoma and Lewis lung carcinoma murine tumor models (18). Immune suppressive leukocytes, including Tregs sensitive to PI3Kδ inhibition, are believed to play a contributory role in limiting antitumor effectiveness of CPI therapy (16). PI3Kδ inhibition together with PD-L1 blockade enhanced efficacy in a T cell–inflamed model (18). Therefore, we tested the combined inhibition of PD-(L)1 and PI3Kδ.

We conducted a phase Ib platform study to explore the clinical safety and tolerability of increasing doses of either itacitinib or parsaclisib in combination with established dosing of the PD-1 inhibitor pembrolizumab in patients with solid tumors. Paired biomarker analysis of treatments and changes within the TME were also examined to provide insight on incorporating JAK1 or PI3Kδ inhibition.

## Materials and Methods

### Study Design

**FIGURE 1:**
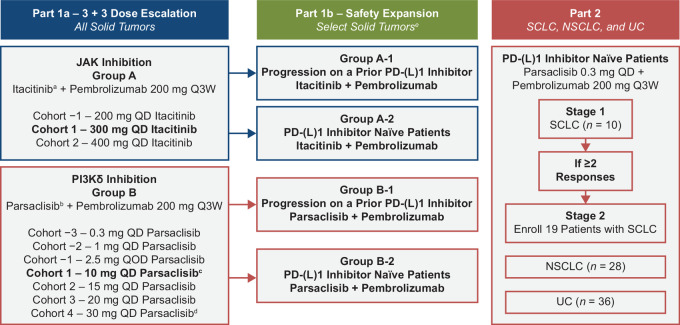
Study design. Note: As of Protocol Amendment 8, enrollment was closed before reaching the planned number of patients. ^a^Cohort 1 was initially evaluated. Cohort −1 was evaluated if the Cohort 1 dose proved intolerable. ^b^Cohort 1 was initially evaluated. Further dose exploration may be evaluated on the basis of emerging pharmacokinetic/pharmacodynamic or safety data. Subsequent increases in the dose of parsaclisib were limited to ≤50% and did not exceed the dose level tested as monotherapy. ^c^Patients treated at ≥10 mg switched to a once weekly dosing schedule at cycle 4 day 1 and beyond. ^d^Based on the review of safety data from the parsaclisib program, patients receiving 30 mg once daily of parsaclisib had the dose reduced to 20 mg once daily. ^e^Selected solid tumor types included endometrial cancer, gastric cancer, HNSCC, melanoma, MSI-CRC or other mismatch repair–deficient tumors, NSCLC, PDAC, RCC, TNBC, or UC. CRC, colorectal cancer; HNSCC, head and neck squamous cell carcinoma; MSI, microsatellite instability; NSCLC, non–small cell lung cancer; PD-(L)1, programmed cell death-(ligand)1; PDAC, pancreatic ductal adenocarcinoma; Q3W, every 3 weeks; QD, once daily; QOD, every other day; RCC, renal cell carcinoma; SCLC, small cell lung cancer; TNBC, triple-negative breast cancer; UC, urothelial carcinoma.

This phase Ib, multicenter, open-label, platform study (ClinicalTrials.gov identifier: NCT02646748) was conducted in the United States. The study evaluated combination of itacitinib or parsaclisib plus pembrolizumab in two parts: Part 1 consisted of Part 1a dose-escalation (enrolling all solid tumors) and Part 1b safety-expansion (focused on selected solid tumors); and Part 2 evaluated parsaclisib plus pembrolizumab in patients with small cell lung cancer (SCLC), NSCLC, and UC (Fig. 1).

Dose-escalation (Part 1a) was performed using a 3+3 design to evaluate the MTD or pharmacologically active dose (PAD) for itacitinib or parsaclisib, in combination with a fixed dose of pembrolizumab (200 mg intravenously every 3 weeks). The highest dose at which less than one-third of patients experienced a dose-limiting toxicity (DLT) was defined as the MTD. Initial combination doses were based on previous results with itacitinib plus nab-paclitaxel and gemcitabine in advanced solid tumors (19) or parsaclisib monotherapy in relapsed or refractory B-cell malignancies (20). In Group A, patients were treated with itacitinib once daily (starting at 300 mg, decreasing to 200 mg if intolerable, or increasing to 400 mg) orally. In Group B, patients were treated for cycles 1–3 with oral parsaclisib once daily (starting at 10 mg, decreasing to 1 or 0.3 mg if intolerable, or increasing to 15, 20, or 30 mg), or every other day (2.5 mg); beginning on cycle 4 day 1 patients were treated with oral parsaclisib once daily (0.3 or 1 mg), every other day (2.5 mg), or once weekly (10, 15, or 20 mg). Safety-expansion (Part 1b) for Group A and B was conducted at doses selected in dose-escalation (Part 1a); Group A-1 (itacitinib plus pembrolizumab) and B-1 (parsaclisib plus pembrolizumab) enrolled patients with prior history of PD-(L)1–targeted therapy, and Group A-2 (itacitinib plus pembrolizumab) and B-2 (parsaclisib plus pembrolizumab) enrolled patients who were PD-(L)1 treatment-naïve (Fig. 1).

In Part 2 (parsaclisib plus pembrolizumab), PD-(L)1 treatment-naïve patients were enrolled and received parsaclisib (0.3 mg once daily) plus pembrolizumab (200 mg every 3 weeks) combination (Fig. 1).

If pembrolizumab treatment was discontinued, treatment with itacitinib or parsaclisib was also discontinued and the patient entered follow-up. If itacitinib or parsaclisib treatment was discontinued, treatment with pembrolizumab could continue after consultation with the sponsor.

### Patient Inclusion and Exclusion Criteria

The study enrolled men and women ≥18 years of age with an Eastern Cooperative Oncology Group performance status (ECOG PS) ≤1, who were willing to provide a baseline and on-treatment tumor biopsy specimen, and had measurable disease based on RECIST v1.1. In Part 1a, enrolled patients had histologically or cytologically confirmed advanced or metastatic solid tumors that progressed after previous standard therapy. In Part 1b, patients enrolled were PD-(L)1 treatment-naïve or had disease progression on prior PD-(L)1–targeted therapy, and had histologically or cytologically confirmed advanced or metastatic endometrial cancer, gastric cancer, melanoma, microsatellite unstable colorectal cancer or other mismatch repair–deficient tumors, NSCLC, HNSCC, renal cell carcinoma, triple-negative breast cancer, genitourinary tract transitional cell carcinoma, or pancreatic ductal adenocarcinoma. In Part 2, patients who were PD-(L)1 treatment-naïve with histologically or cytologically confirmed advanced or metastatic SCLC, NSCLC, or UC were enrolled.

Key exclusion criteria were serum creatinine >1.5 × institutional upper limit of normal (ULN), alkaline phosphatase, aspartate and/or alanine aminotransferase ≥2.5 × ULN, or total bilirubin ≥1.5 × ULN, persistent grade >1 toxic effects from prior therapy, or received live vaccine within 30 days. Additional exclusion criteria included having active infection (requiring systemic therapy), autoimmune disease, or central nervous system metastases and/or carcinomatous meningitis; current or history of pneumonitis; abnormal electrocardiogram; history of interstitial lung disease; history of human immunodeficiency virus infection; or evidence of hepatitis B or C virus infection or risk of reactivation. Patients who presented with unresolved (grade ≥2) toxicities from previous therapy and/or surgical complications were excluded from the study. All suspected immune-related adverse events (AE) during the study were treated with appropriate supportive care measures as determined by the treating investigator.

### Study Endpoints and Assessments

The primary study endpoint was safety and tolerability in Part 1, assessed by monitoring the frequency, duration, and severity of AEs. A treatment-emergent adverse event (TEAE) was defined as an AE either reported for the first time or worsening of a preexisting event after first dose of study drug. AEs were classified into system organ class and Medical Dictionary for Regulatory Activities preferred term, and severity of AEs was assessed using Common Terminology Criteria for Adverse Events (v4.03). Secondary endpoints in Part 1 and 2 were ORR determined by radiographic disease assessments per RECIST v1.1, and change in the number of tumor-infiltrating lymphocytes (TIL) and ratio of CD8^+^ effector T cell (Teff) to forkhead box protein 3 (FoxP3)^+^ (Treg) tumor-infiltrating cells (on-treatment vs. baseline) assessed by IHC.

Exploratory study endpoints in Part 1 and 2 were pharmacokinetics of itacitinib and parsaclisib, and biomarker effects in plasma and tumor tissue after treatment with itacitinib or parsaclisib in combination with pembrolizumab. Plasma samples for pharmacokinetic analysis were collected predose on cycle 1 day 1, day 8 (±3 days), day 15 (±3 days), and cycle 2 day 1 (±3 days). On day 1 of cycle 1 and 2, plasma samples were also collected at 60 (±10) minutes, 2 (±0.5) hours, 4 (±0.5) hours, and 6 (±1) hours postdose. Plasma concentrations of itacitinib or parsaclisib were determined using validated LC/MS-MS method (Incyte Research Corporation; refs. 21, 22), and pharmacokinetic calculations were performed using standard noncompartmental methods with Phoenix WinNonlin (Certara USA Inc, v8.2). Tumor biopsy samples were collected at baseline (before day 1 administration) and on-treatment (between weeks 5 and 6 following second dose of pembrolizumab) for translational analysis.

### Translational Biomarker Analyses

Changes in TILs and the ratio of Teff to Tregs (CD8^+^:FoxP3^+^) from baseline and on-treatment tumor biopsies were analyzed to investigate effects of the different treatment regimens on the TME. In Parts 1 and 2, the 22C3 pharmDx (Agilent/Dako) chromogenic IHC assay for PD-L1 expression was performed on biopsy samples at Indivumed. Membranous anti-PD-L1 staining was semiquantitatively evaluated using the histo (H)-score and tumor proportion score (TPS; TPS low >1 to ≤49%; TPS high ≥50%).

In Part 1, a single-color, Singleplex chromogenic assay (Indivumed) was used to quantify cells in the combined tumor plus stromal (nontumor) regions in baseline and on-treatment biopsy samples. In Part 2, a five-color, Multiplex IHC assay (Indivumed) was used to quantify cell densities separately in the tumor and stromal regions, in addition to a composite of the total tissue section from baseline and on-treatment biopsy samples. The Multiplex assay measured CD3^+^, CD8^+^, FoxP3^+^, panCK^+^ (pan-cytokeratin) or pMEL^+^ (premelanosome) cells, and nuclear counterstain DAPI (4′,6-diamidino-2-phenylindole). Cell densities (cells/mm^2^) for both Singleplex and Multiplex IHC assays were determined by digital image analysis at OracleBio.

### Plasma Protein Analysis

To further evaluate the pharmacodynamic effects and potential association with clinical response to study treatment, the presence of immune and nonimmune plasma proteins was determined using a Multiplex Proximity Extension Assay by Olink Proteomics. In this assay, each biomarker is identified by a matched pair of antibodies coupled to unique, partially complementary oligonucleotides and measured by qRT-PCR, with more than 1,100 plasma analytes evaluated per sample.

### Statistical Analysis

All patients enrolled in the study who received ≥1 dose of study drug (pembrolizumab, itacitinib, or parsaclisib) comprised the safety analysis population and the full analysis set (FAS) for baseline demographic and efficacy analyses. Descriptive statistics were used to summarize continuous and categorical variables. The exact method for binomial distributions was used to calculate the 95% confidence interval (CI) of ORR. The Kaplan–Meier method was used to estimate duration of response and progression-free survival, including median value and 95% CI.

The pharmacokinetic and pharmacodynamic evaluable population included all patients in the FAS who provided at ≥1 plasma sample (≥1 pharmacokinetic or pharmacodynamic measurement). Summary statistics were calculated for pharmacokinetic parameters of itacitinib and parsaclisib, and analysis of pharmacodynamic data. The Wilcoxon matched-pairs signed-rank test (GraphPad Prism v7.02, GraphPad Software) was used to compare baseline and on-treatment tumor biopsy samples, with changes in TILs deemed significant at *P* value <0.05.

In Part 1a, 3 to 6 patients were enrolled in each dose level depending on the occurrence of DLTs. In Part 1b, with a planned enrollment of 60 patients [30 patients per expansion cohort A-1/A-2 (itacitinib plus pembrolizumab) and B-1/B-2 (parsaclisib plus pembrolizumab)], there was a ≥90% chance of observing a toxicity with a true event rate of >7.4%. In Part 2, with planned enrollment of a total of 10–29 patients with SCLC in a Simon two-stage design, and 28 patients with NSCLC and 36 patients with UC in a Simon one-stage design, the cohorts were to be terminated for lack of efficacy if there were one or fewer SCLC in stage 1 or five or fewer SCLC total in stage 1 and 2, seven or fewer NSCLC, or ≤13 patients with UC responded to treatment. This was an exploratory study and no formal statistical tests were performed.

Effects of treatment on plasma protein biomarkers were determined by paired *t* test comparing baseline (cycle 1 day 1) values to on-treatment values (cycle 2, 4, and/or 6 day 1). A difference was deemed significant at a FDR *P* value <0.05 and a log_2_ fold change of >0.4 or ≤0.4.

### Data Availability Statement

Access to individual patient-level data is not available for this study.

### Ethics Statement

The study was performed in accordance with the International Council for Harmonisation Guideline for Good Clinical Practice, the principles of the Declaration of Helsinki, and other applicable local ethical and legal requirements. The study protocol and its amendments were reviewed and approved by institutional review boards or independent ethics committees, and patients provided written informed consent before enrollment.

## Results

### Patient Characteristics and Disposition

At the data cut-off date (March 10, 2020), a total of 159 patients had been enrolled at 11 study sites and were included in both the safety population and FAS. Forty-nine patients were included in all of Part 1/Group A [itacitinib plus pembrolizumab; *n* = 8 in Part 1a/Group A, *n* = 41 in Part 1b/Groups A-1 (PD-(L)1 treatment-experienced) and A-2 (PD-(L)1 treatment-naïve)]; 83 patients were included in all of Part 1/Group B [parsaclisib plus pembrolizumab; *n* = 34 in Part 1a/Group B, *n* = 49 in Part 1b/Groups B-1 (PD-(L)1 treatment-experienced) and B-2 (PD-(L)1 treatment-naïve)]; and 27 patients with SCLC, NSCLC, or UC were included in Part 2 (parsaclisib plus pembrolizumab). On the basis of the limited efficacy observed in Part 1b/Group A-1/A-2, Part 2 did not include an itacitinib plus pembrolizumab treatment combination.

Patient demographics and baseline characteristics are summarized in Table 1. Median (range) patient age was 64.5 (46–74) years in Part 1a/Group A, 63.0 (33–79) years in Part 1a/Group B, 66.0 (33–83) years in Part 1b/Group A-1/A-2, 66.0 (29–80) years in Part 1b/Group B-1/B-2, and 66.0 (55–89) years in Part 2. In Part 1 and 2, >50% of patients in each treatment group had an ECOG PS of 1 at baseline and ≥60% of patients had received systemic therapy before enrolling in the study.

**TABLE 1:** Summary of demographic and baseline characteristics

	Part 1: Itacitinib + Pembrolizumab or Parsaclisib + Pembrolizumab				Part 2: Parsaclisib + Pembrolizumab			
Variable	Part 1a Group A (itacitinib) (*n* = 8)	Part 1a Group B (parsaclisib) (*n* = 34)	Part 1b Group A-1/A-2 (itacitinib) (*n* = 41)	Part 1b Group B-1/B-2 (parsaclisib) (*n* = 49)	SCLC^a^ 0.3 mg QD/200 mg Q3W (*n* = 14)	NSCLC 0.3 mg QD/200 mg Q3W (*n* = 8)	UC 0.3 mg QD/200 mg Q3W (*n* = 5)	Part 2 Total (*n* = 27)
Median age (range), years	64.5 (46–74)	63.0 (33–79)	66.0 (33–83)	66.0 (29–80)	64.5 (57–89)	70.0 (58–82)	62.0 (55–87)	66.0 (55–89)
Male, *n* (%)	3 (37.5)	15 (44.1)	18 (43.9)	27 (55.1)	5 (35.7)	3 (37.5)	3 (60.0)	11 (40.7)
Race, *n* (%)								
White	7 (87.5)	29 (85.3)	38 (92.7)	42 (85.7)	14 (100.0)	8 (100.0)	3 (60.0)	25 (92.6)
African American	1 (12.5)	2 (5.9)	3 (7.3)	2 (4.1)	0	0	0	0
Asian	0	2 (5.9)	0	3 (6.1)	0	0	2 (40.0)	2 (7.4)
Other	0	1 (2.9)	0	2 (4.1)	0	0	0	0
ECOG PS, *n* (%)								
0	1 (12.5)	10 (29.4)	15 (36.6)	24 (49.0)	2 (14.3)	0	0	2 (7.4)
1	7 (87.5)	24 (70.6)	26 (63.4)	25 (51.0)	12 (85.7)	8 (100.0)	5 (100.0)	25 (92.6)
Prior therapy, *n* (%)								
Systemic therapy	8 (100)	32 (94.1)	39 (95.1)	46 (93.9)	12 (85.7)	5 (62.5)	3 (60.0)	20 (74.1)
Surgery	8 (100)	31 (91.2)	37 (90.2)	40 (81.6)	11 (78.6)	6 (75.0)	5 (100.0)	22 (81.5)
Radiotherapy	3 (37.5)	21 (61.8)	24 (58.5)	22 (44.9)	9 (64.3)	2 (25.0)	0 (0.0)	11 (40.7)
PD-L1 status, *n* (%)								
Positive	0	2 (5.9)	2 (4.9)	2 (4.1)	1 (7.1)	1 (12.5)	2 (40.0)	4 (14.8)
Negative	0	1 (2.9)	3 (7.3)	5 (10.2)	1 (7.1)	7 (87.5)	0	8 (29.6)
Unknown	0	0	0	1 (2.0)	0	0	0	0
Missing	8 (100)	31 (91.2)	36 (87.8)	41 (83.7)	12 (85.7)	0	3 (60.0)	15 (55.6)
Tumor type, *n* (%)								
Endometrial adenocarcinoma	0	2 (5.9)	2 (4.9)	5 (10.2)	–	–	–	–
Bladder cancer	0	0	0	6 (12.2)	–	–	5 (100.0)	5 (18.5)
Breast cancer	1 (12.5)	2 (5.9)	3 (7.3)	3 (6.1)	–	–	–	–
CRC	2 (25)	4 (11.8)	1 (2.4)	1 (2.0)	–	–	–	–
Gastric	0	0	1 (2.4)	2 (4.1)	–	–	–	–
HNSCC	0	1 (2.9)	3 (7.3)	5 (10.2)	–	–	–	–
Melanoma	0	4 (11.8)	11 (26.8)	9 (18.4)	–	–	–	–
NSCLC	3 (37.5)	6 (17.6)	7 (17.1)	8 (16.3)	–	8 (100.0)	–	8 (29.6)
Pancreatic	1 (12.5)	1 (2.9)	8 (19.5)	4 (8.2)	–	–	–	–
RCC	0	1 (2.9)	4 (9.8)	4 (8.2)	–	–	–	–
SCLC	0	0	0	0	14 (100.0)	–	–	14 (51.9)
Solid tumor	1 (12.5)	13 (38.2)	1 (2.4)	2 (4.1)	–	–	–	–

Abbreviations: CRC, colorectal cancer; ECOG PS, Eastern Cooperative Oncology Group Performance Status; HNSCC, head and neck squamous cell carcinoma; NSCLC, non–small cell lung cancer; PD-L1, programmed death-ligand 1; Q3W, every 3 weeks; QD, once daily; RCC, renal cell carcinoma; SCLC, small cell lung cancer; UC, urothelial carcinoma.

^a^Included one patient who received parsaclisib at a starting dose of 20 mg once daily.

All 49 patients in Part 1a/Group A and Part 1b/Groups A-1/A-2 (itacitinib plus pembrolizumab), 74 (89.2%) patients in Part 1a/Group B and Part 1b/Groups B-1/B-2 (parsaclisib plus pembrolizumab), and 26 (96.3%) patients in Part 2 (parsaclisib plus pembrolizumab) discontinued treatment. Progressive disease (PD) was the most common reason for discontinuation in Part 1a/Group A [6 (75%)] and B [16 (47.1%)], Part 1b/Group A-1/A-2 [29 (70.7%)] and B-1/B-2 [30 (61.2%)], and Part 2 [13 (48.1%)]. AEs were also a common reason for treatment discontinuation of pembrolizumab in Part 1a/Group B [14 (41.2%)]. No patients remain on the study. Detailed patient disposition for each dose level evaluated in different treatment groups in Parts 1 and 2 are provided in Supplementary Tables S1–S5.

### Safety

Itacitinib 300 mg once daily was selected as the MTD in dose-escalation Part 1a/Group A and used in safety-expansion Part 1b/Group A-1 [PD-(L)1 treatment-experienced] and A-2 [PD-(L)1 treatment-naïve]. Parsaclisib 30 mg once daily was selected as the MTD/PAD in dose-escalation Part 1a/Group B, and parsaclisib 20 mg once daily and 30 mg once daily were selected for safety-expansion Part 1b/Group B-1 [PD-(L)1 treatment-experienced] and B-2 [PD-(L)1 treatment-naïve]. Taking into consideration that higher doses of parsaclisib (20 or 30 mg once daily) required prophylaxis for *Pneumocystis jirovecii* pneumonia (PJP), which was difficult for patients to tolerate, and following *in vitro* evaluation of pharmacodynamic effects of parsaclisib on Treg and Teff biology that suggested the parsaclisib 20–30 mg once daily dose administered in Part 1 could also be inhibiting Teff function, a parsaclisib 0.3 mg once daily dose was selected for all patient groups in Part 2.

#### Part 1: Itacitinib plus Pembrolizumab

No patient in Part 1a/Group A, or Part 1b/Group A-1 [PD-(L)1 treatment-experienced] or A-2 [PD-(L)1 treatment-naïve] experienced a DLT. All patients in Part 1 treated with itacitinib experienced ≥1 TEAE (Supplementary Table S6). Itacitinib treatment-related adverse events (TRAE) were reported by 5 (62.5%) patients in Part 1a/Group A; the most common were fatigue [3 (37.5%)], and anemia, decreased appetite and nausea [each 2 (25.0%)] (Table 2). Itacitinib TRAEs occurred in 31 (75.6%) patients in Part 1b/Group A-1/A-2; the most common being fatigue [9 (22.0%)], nausea [8 (19.5%)], anemia and diarrhea [each 6 (14.6%)] (Table 2).

**TABLE 2:** Summary of itacitinib and parsaclisib treatment-related TEAEs by MedDRA preferred term (≥5% of patients in the safety population)

	Part 1: Itacitinib + Pembrolizumab or Parsaclisib + Pembrolizumab				Part 2: Parsaclisib + Pembrolizumab			
Preferred term, *n* (%)	Part 1a Group A (itacitinib) (*n* = 8)	Part 1a Group B (parsaclisib) (*n* = 34)	Part 1b Group A-1/A-2 (itacitinib) (*n* = 41)	Part 1b Group B-1/B-2 (parsaclisib) (*n* = 49)	SCLC^a^ 200 mg Q3W/0.3 mg QD (*n* = 14)	NSCLC 200 mg Q3W/0.3 mg QD (*n* = 8)	UC 200 mg Q3W/0.3 mg QD (*n* = 5)	Part 2 Total (*n* = 27)
Any treatment-related TEAE	6 (75.0)	29 (85.3)	32 (78.0)	37 (75.5)	10 (71.4)	6 (75.0)	3 (60.0)	19 (70.4)
Fatigue	3 (37.5)	11 (32.4)	9 (22.0)	12 (24.5)	5 (35.7)	1 (12.5)	1 (20.0)	7 (25.9)
Anemia	2 (25.0)	2 (5.9)	6 (14.6)	1 (2.0)	0	0	1 (2.0)	1 (3.7)
Decreased appetite	2 (25.0)	5 (14.7)	3 (7.3)	2 (4.1)	1 (7.1)	1 (12.5)	0	2 (7.4)
Nausea	2 (25.0)	8 (23.5)	8 (19.5)	12 (24.5)	2 (14.3)	1 (12.5)	0	3 (11.1)
Alopecia	1 (12.5)	0	0	0	0	0	0	0
Aspartate aminotransferase increased	1 (12.5)	4 (11.8)	1 (2.4)	1 (2.0)	0	0	0	0
Dysgeusia	1 (12.5)	0	1 (2.4)	5 (10.2)	0	0	0	0
Early satiety	1 (12.5)	0	0	0	0	0	0	0
Leukopenia	1 (12.5)	1 (2.9)	0	0	0	1 (12.5)	0	1 (3.7)
Platelet count decreased	1 (12.5)	1 (2.9)	1 (2.4)	1 (2.0)	0	0	0	0
Vomiting	1 (12.5)	4 (11.8)	2 (4.9)	4 (8.2)	0	1 (12.5)	0	1 (3.7)
Weight decreased	1 (12.5)	4 (11.8)	0	3 (6.1)	1 (7.1)	1 (12.5)	0	2 (7.4)
Diarrhea	0	7 (20.6)	6 (14.6)	6 (12.2)	2 (14.3)	1 (12.5)	0	3 (11.1)
Pyrexia	0	6 (17.6)	4 (9.8)	9 (18.4)	1 (7.1)	0	0	1 (3.7)
Pruritus	0	5 (14.7)	1 (2.4)	4 (8.2)	2 (14.3)	0	1 (20.0)	3 (11.1)
Maculopapular rash	0	5 (14.7)	1 (2.4)	0	3 (21.4)	0	1 (20.0)	4 (14.8)
Alanine aminotransferase increased	0	4 (11.8)	2 (4.9)	0	0	0	0	0
Chills	0	3 (8.8)	1 (2.4)	1 (2.0)	0	0	0	0
Hypokalemia	0	3 (8.8)	0	2 (4.1)	0	0	0	0
Arthralgia	0	2 (5.9)	1 (2.4)	4 (8.2)	1 (7.1)	0	0	1 (3.7)
Colitis	0	2 (5.9)	0	1 (2.0)	0	0	0	0
Dizziness	0	2 (5.9)	0	2 (4.1)	0	0	0	0
Dry skin	0	2 (5.9)	0	0	0	0	0	0
Flatulence	0	2 (5.9)	1 (2.4)	0	0	0	0	0
Hypotension	0	2 (5.9)	0	0	1 (7.1)	0	0	1 (3.7)
Pneumonitis	0	2 (5.9)	0	3 (6.1)	1 (7.1)	0	0	1 (3.7)
Rash	0	2 (5.9)	2 (4.9)	6 (12.2)	0	0	1 (20.0)	1 (3.7)
Thrombocytopenia	0	2 (5.9)	1 (2.4)	0	0	1 (12.5)	0	1 (3.7)
Constipation	0	1 (2.9)	3 (7.3)	1 (2.0)	1 (7.1)	0	0	1 (3.7)
Neutrophil count decreased	0	0	0	6 (12.2)	1 (7.1)	1 (12.5)	0	2 (7.4)
Abdominal pain	0	0	1 (2.4)	4 (8.2)	1 (7.1)	0	0	1 (3.7)
Dry mouth	0	0	1 (2.4)	3 (6.1)	1 (7.1)	0	0	1 (3.7)
Headache	0	1 (2.9)	1 (2.4)	3 (6.1)	0	1 (12.5)	0	1 (3.7)
Stomatitis	0	0	0	3 (6.1)	0	0	0	0
White blood cell count decreased	0	1 (2.9)	0	3 (6.1)	1 (7.1)	1 (12.5)	0	2 (7.4)
Dysphagia	0	0	1 (2.4)	0	2 (14.3)	0	0	2 (7.4)
Muscle spasms	0	0	0	1 (2.0)	1 (7.1)	1 (12.5)	0	2 (7.4)
Muscular weakness	0	0	0	0	2 (14.3)	0	0	2 (7.4)

Abbreviations: MedDRA, Medical Dictionary for Regulatory Activities; NSCLC, non-small cell lung cancer; Q3W, every 3 weeks; QD, once daily; SCLC, small cell lung cancer; TEAE, treatment-emergent adverse event; UC, urothelial carcinoma.

^a^Included one patient who received parsaclisib at a starting dose of 20 mg once daily.

A total of 26 patients experienced ≥1 serious TEAE [6 (75.0%)] in Part 1a/Group A, and 20 (48.8%) in Part 1b/Group A-1/A-2 (Supplementary Table S7). Serious TEAEs reported in ≥2 patients overall in Part 1a/Group A were pyrexia and back pain [2 each (25.0%)], and in Part 1b/Group A-1/A-2 were pyrexia, urinary tract infection, and pulmonary embolism [3 each (7.3%)], and gastric obstruction and pneumonia [2 each (4.9%)]. Disease progression could also be reported as a serious TEAE per the study protocol, and occurred in 8 (19.5%) patients in Part 1b/Group A-1/A-2.

Serious TRAEs considered related to pembrolizumab were reported in one patient in Part 1b/Group A-1 [chills and pyrexia (each 2.4%)], and considered related to itacitinib were reported in 2 patients in Part 1b/Group A-1/A-2 [pyrexia in 2 (4.9%) patients, and chills in 1 (2.4%) patient]. No serious TEAE reported in Part 1a/Group A were considered related to either pembrolizumab or itacitinib. Two patients in Part 1b/Group A-2 [PD-(L)1 treatment-naïve] had TEAEs of special interest, with laboratory results that met the criteria for potential drug-induced liver injury; neither patient met Hy’s Law criteria. TEAEs of grade ≥3 were reported in 7 patients (87.5%) in Part 1a/Group A and in 27 (65.9%) of patients in Part 1b/Group A-1/A2.

Treatment was discontinued in 1 patient (12.5%) in Part 1a/Group A because of a TEAE of back pain. Both itacitinib and pembrolizumab were discontinued in 10 patients (24.4%) in Part 1b (2 patients in Group A-1 and 8 patients in Group A-2) because of TEAEs; 4 of the 10 patients discontinued because of malignant neoplasm progression. In Part 1b/Group A-2, 1 patient had itacitinib discontinued on day 153 (because of pyrexia), and pembrolizumab discontinued on day 184 (because of bilateral pulmonary emboli associated with progression of primary pancreatic carcinoma).

#### Part 1: Parsaclisib plus Pembrolizumab

One 72-year-old patient with NSCLC in Part 1a/Group B (receiving parsaclisib 2.5 mg once daily plus pembrolizumab) experienced two grade 4 DLTs of pneumonitis (day 12, considered related to pembrolizumab and parsaclisib; not resolved) and oliguria (day 14, considered related to parsaclisib; fatal). No patient in Part 1b/Group B-1 [PD-(L)1 treatment-experienced] or B-2 [PD-(L)1 treatment-naïve] experienced a DLT. All patients in Part 1 treated with parsaclisib experienced ≥1 TEAE (Supplementary Table S6). Parsaclisib TRAEs were reported in 24 (70.6%) patients in Part 1a/Group B, the most common were fatigue [11 (32.4%)], nausea [8 (23.5%)], diarrhea [7 (20.6%)], and pyrexia [6 (17.6%)] (Table 2). Parsaclisib TRAEs occurred in 36 (73.5%) patients in Part 1b/Group B-1/B-2; the most common events were fatigue and nausea [each 12 (24.5%)], pyrexia [9 (18.4%)], diarrhea, neutrophil count decreased and rash [each 6 (12.2%)] (Table 2).

A total of 50 patients experienced ≥1 serious TEAE [23 (67.6%) in Part 1a/Group B, 27 (55.1%) in Part 1b/Group B-1/B-2] (Supplementary Table S7). Serious TEAEs reported in ≥2 patients in Part 1a/Group B included pneumonia and urinary tract infection [each 4 (11.8%)], small intestinal obstruction, sepsis, malignant neoplasm progression, and pneumonitis [each 2 (5.9%)], and in Part 1b/Group B-1/B-2 were atrial fibrillation, sepsis, and malignant neoplasm progression [each 3 (6.1%)], dehydration, acute kidney injury, hypoxia, and respiratory failure [each 2 (4.1%)]. Serious TRAEs reported in Part 1a/Group B were pneumonitis [2 (5.9%)], hemolytic anemia and colitis [each one (2.9%)], with additional serious TRAEs of pneumonia and oliguria [each one (2.9%)] considered related only to parsaclisib. In Part 1b/Group B-1/B-2 serious TRAEs were gastritis, diverticulitis, and confusional state [each one (2.0%)], with additional serious TRAEs of dehydration, nausea, vomiting, and PJP [each one (2.0%)] attributed only to parsaclisib. Grade ≥3 TEAEs were reported in 27 patients (79.4%) in Part 1a/Group B and in 34 (69.4%) of patients in Part 1b/Group B-1/B2.

Fifteen patients (44.1%) in Part 1a/Group B experienced TEAEs leading to discontinuation of parsaclisib, and 14 patients (41.2%) had TEAEs leading to discontinuation of pembrolizumab. TEAEs leading to treatment discontinuation occurred in 1 (2.9%) patient each except for increased alanine aminotransferase, increased aspartate aminotransferase, and depressed level of consciousness [each 2 (5.9%)]. Nine patients (18.4%) in Part 1b/Group B-1/B-2 experienced TEAEs leading to discontinuation of parsaclisib, and 8 patients (16.3%) had TEAEs leading to discontinuation of pembrolizumab.

#### Part 2: Parsaclisib plus Pembrolizumab

No DLTs were reported in patients enrolled in Part 2. All patients experienced ≥1 TEAE (Supplementary Table S6). Parsaclisib TRAEs were reported by 16 (59.3%) patients in Part 2; fatigue [7 (25.9%)], maculopapular rash [4 (14.8%)], diarrhea, nausea, and pruritus [each 3 (11.1%)] were the most common events (Table 2). Sixteen (59.3%) patients in Part 2 experienced ≥1 serious TEAE (Supplementary Table S7); serious TEAEs reported in ≥1 patient were pneumonia [6 (22.2%)], metastatic SCLC (progression of primary SCLC), acute respiratory failure, pleural effusion, and pneumothorax [each 2 (7.4%)]. Serious TRAEs included anaphylactic reaction [one (3.7%)] considered related to parsaclisib, dysphagia, and pneumonia [one each (3.7%)] considered related to parsaclisib and pembrolizumab, and acute pancreatitis [one (3.7%)] considered related to pembrolizumab. A 59-year-old patient with SCLC was diagnosed with myelodysplastic syndrome considered by the investigator to be serious (medically significant) and possibly related to parsaclisib; however, there was insufficient information to meaningfully assess causality as patients with lung cancer are at risk of secondary hematologic malignancies. Grade ≥3 TEAEs were reported in 20 patients (74.1%) in Part 2.

Nine patients (33.3%) in Part 2 experienced TEAEs leading to discontinuation of parsaclisib, and 8 patients (29.6%) had TEAEs leading to discontinuation of pembrolizumab.

### Efficacy

Of patients receiving itacitinib plus pembrolizumab in Part 1, 4 (8.2%) had partial response (PR) and 19 (38.8%) had stable disease (SD; Table 3). In Part 1a/Group A, 5 (62.5%) patients had SD. In Part 1b/Group A-1 [PD-(L)1 therapy-experienced], 1 (5.0%) patient achieved a PR (squamous NSCLC) for an ORR of 5.0% (95% CI, 0.00–0.25), and an additional 10 (50.0%) patients had SD as their best response. In Part 1b/Group A-2 [PD-(L)1 treatment-naïve], 3 (14.3%) patients achieved a PR (NSCLC adenocarcinoma, pancreatic cancer, renal cell carcinoma, *n* = 1 each) for an ORR of 14.3% (95% CI, 0.03–0.36), and 4 (19.0%) patients had SD.

**TABLE 3:** Tumor response by RECIST (full analysis set)

	Part 1: Itacitinib + Pembrolizumab or Parsaclisib + Pembrolizumab				Part 2: Parsaclisib + Pembrolizumab			
Variable	Part 1a Group A (itacitinib) (*n* = 8)	Part 1a Group B (parsaclisib) (*n* = 34)	Part 1b Group A-1/A-2 (itacitinib) (*n* = 41)	Part 1b Group B-1/B-2 (parsaclisib) (*n* = 49)	SCLC^a^ 0.3 mg QD/200 mg Q3W (*n* = 14)	NSCLC 0.3 mg QD/200 mg Q3W (*n* = 8)	UC 0.3 mg QD/200 mg Q3W (*n* = 5)	Part 2 Total (*n* = 27)
Best overall response, *n* (%)								
Complete response	0	1 (2.9)^b^	0	4 (8.2)^c^	0	0	0	0
Partial response	0	6 (17.6)^d^	4 (9.8)^e^	3 (6.1)^f^	3 (21.4)	2 (25.0)	0	5 (18.5)
Stable disease	5 (62.5)	10 (29.4)	14 (34.1)	11 (22.4)	1 (7.1)	4 (50.0)	1 (20.0)	6 (22.2)
Progressive disease	3 (37.5)	11 (32.4)	16 (39.0)	27 (55.1)	7 (50.0)	2 (25.0)	4 (80.0)	13 (48.1)
Not assessed/evaluable	0	6 (17.6)	7 (17.1)	4 (8.2)	3 (21.4)	0	0	3 (11.1)
Objective response, *n* (%)^g^	0	7 (20.6)	4 (9.8)	7 (14.3)	3 (21.4)	2 (25.0)	0	5 (18.5)
95% CI for ORR^h^	(0.00–0.37)	(0.09–0.38)	(0.03–0.23)	(0.06–0.27)	(0.05–0.51)	(0.03–0.65)	(0.00–0.52)	(0.06–0.38)

Abbreviations: CI, confidence interval; NSCLC, non–small cell lung cancer; ORR, objective response rate; Q3W, every 3 weeks; QD, once daily; SCLC, small cell lung cancer; UC, urothelial carcinoma.

^a^Included one patient who received parsaclisib at a starting dose of 20 mg once daily.

^b^NSCLC (metastatic liver).

^c^Bladder cancer, *n* = 2 (metastatic lung, lymph nodes); endometrial adenocarcinoma, *n* = 1 (metastatic lymph nodes); melanoma, *n* = 1 (metastatic lymph nodes).

^d^Adenocarcinoma, *n* = 1 (metastatic lymph nodes); endometrial adenocarcinoma, *n* = 1; NSCLC, *n* = 1 (metastatic lung); cholangiocarcinoma, *n* = 1 (metastatic lymph nodes); SCLC, *n* = 2 (metastatic lymph nodes; metastatic liver, lymph nodes).

^e^NSCLC, *n* = 2 [adenocarcinoma; squamous (metastatic lymph nodes)]; pancreatic cancer, *n* = 1 (metastatic lung, lymph nodes); renal cell carcinoma (clear cell), *n* = 1 (metastatic lung).

^f^Endometrial adenocarcinoma, *n* = 1 (metastatic liver); melanoma, *n* = 1 (metastatic lymph nodes); NSCLC, *n* = 1 (adenocarcinoma; metastatic lymph nodes).

^g^Patients that had best overall response of complete response or partial response.

^h^Confidence intervals were calculated on the basis of the exact method for binomial distributions.

Of patients receiving parsaclisib plus pembrolizumab in Part 1, 5 (6.0%) had complete response (CR), 9 (10.8%) had PR, and 21 (25.3%) had SD (Table 3). In Part 1a/Group B, 1 (2.9%) patient achieved a CR (NSCLC) and 6 (17.6%) patients had a PR (adenocarcinoma, endometrial adenocarcinoma, NSCLC, cholangiocarcinoma, *n* = 1 each; SCLC, *n* = 2) for an ORR of 20.6% (95% CI, 0.09–0.38), and an additional 10 (29.4%) patients had SD as their best response. In Part 1b/Group B-1 [PD-(L)1 therapy-experienced] and B-2 [PD-(L)1 treatment-naïve], a total of 4 (8.2%) patients achieved a CR (bladder cancer, *n* = 2; endometrial adenocarcinoma, *n* = 1; melanoma, *n* = 1) and 3 (6.1%) patients a PR (endometrial adenocarcinoma, melanoma, NSCLC adenocarcinoma, *n* = 1 each) for an ORR of 14.3% (95% CI, 0.06–0.27), and another 11 (22.4%) patients had SD.

Of patients receiving parsaclisib plus pembrolizumab in Part 2 of the study, 5 (18.5%) had PR and 6 (22.2%) had SD (Table 3). Three (21.4%) patients with SCLC achieved a PR for an ORR of 21.4% (95% CI, 0.05–0.51) and 1 (7.1%) patient had SD. Two (25.0%) patients with NSCLC achieved a PR for an ORR of 25.0% (95% CI, 0.03–0.65) and an additional 4 (50.0%) patients had SD. No patients with UC achieved an objective response, and 1 (20.0%) patient had SD as their best response. Enrollment in Part 2 of the study was stopped following ongoing review of efficacy, safety, and biopsy translational data, because observed clinical activity was not considered sufficient to justify study continuation.

### Pharmacokinetics

Itacitinib exposures were higher in patients receiving 300 mg compared with 400 mg once daily (Supplementary Table S8). Given the small number of patients studied at 400 mg once daily, 3 patients at cycle 2 day 1, and the relatively small difference in nominal dose between 300 and 400 mg once daily, this finding is likely because of random variability. Plasma concentrations of itacitinib generally peaked at around 2 hours postdose. Steady state appeared to be reached by cycle 1 day 8. Itacitinib exposures were highly overlapping between patients, regardless of prior PD-1 therapy.

Parsaclisib exposures increased with higher doses (Supplementary Table S9). Parsaclisib reached peak plasma concentrations generally around 1 hour postdose, and steady state appeared to be reached by cycle 1 day 8. Parsaclisib exposures were highly overlapping between patients, irrespective of prior PD-1 therapy.

### Tumor Lymphocyte IHC

**FIGURE 2:**
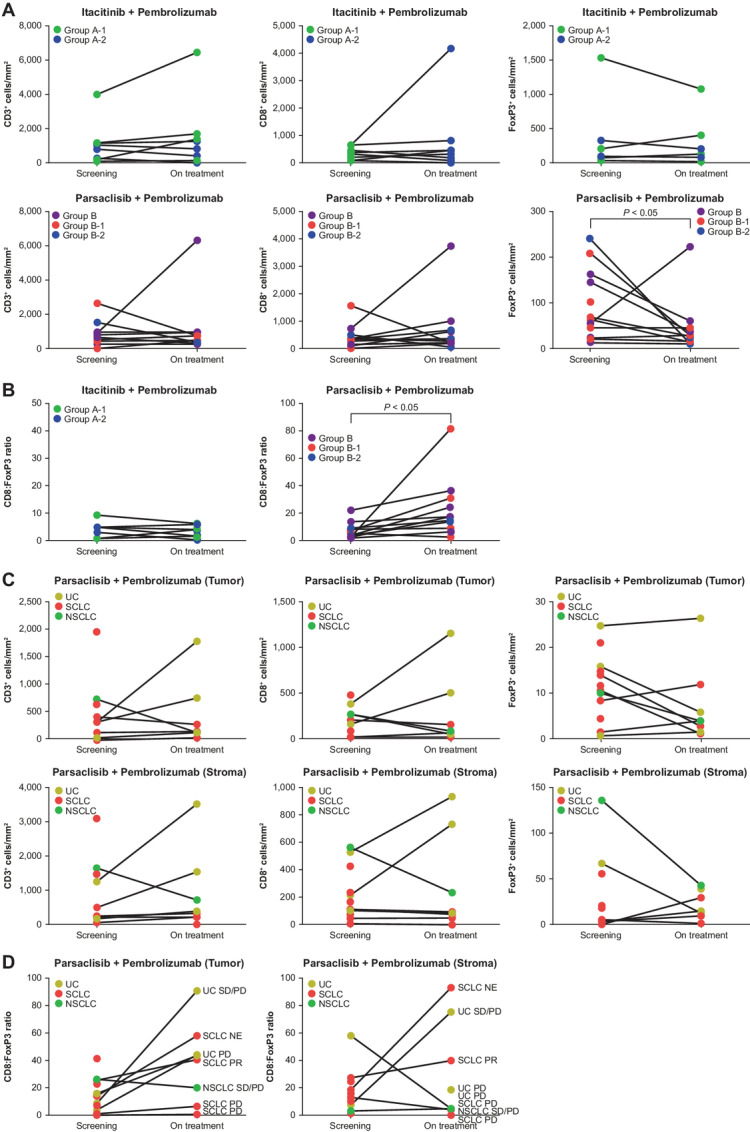
IHC analysis of paired screening and on-treatment (week 5–6) tumor biopsies, from patients receiving itacitinib or parsaclisib in combination with pembrolizumab. **A,** Study Part 1 (T-cell infiltration in combined tumor plus intratumoral stromal regions: CD3^+^, CD8^+^, FoxP3^+^ cells/mm^2^): itacitinib (Group A-1 and A-2, 300 mg once daily) plus pembrolizumab 200 mg every 3 weeks (upper, *n* = 9); parsaclisib (Group B, 0.3–1 mg once daily, or 2.5 mg every other day; B-1 and B-2, 20–30 mg once daily) plus pembrolizumab 200 mg every 3 weeks (lower, *n* = 11). **B,** Study Part 1 (CD8^+^:FoxP3^+^ ratio in combined tumor plus intratumoral stromal regions): itacitinib (300 mg) plus pembrolizumab (left, *n* = 9); parsaclisib (Group B, 0.3–1 mg once daily, or 2.5 mg every other day; B-1 and B-2, 20–30 mg once daily) plus pembrolizumab 200 mg every 3 weeks (right, *n* = 11). **C,** Study Part 2 (T-cell infiltration: CD3^+^, CD8^+^, FoxP3^+^ cells/mm^2^): parsaclisib 0.3 mg once daily plus pembrolizumab (*n* = 8 pairs; NSCLC = 1, SCLC = 4, UC = 3). **D,** Study Part 2 (CD8^+^:FoxP3^+^ ratio): parsaclisib 0.3 mg once daily plus pembrolizumab 200 mg every 3 weeks (*n* = 8 pairs; NSCLC = 1, SCLC = 4, UC = 3). NSCLC, non–small cell lung cancer; SCLC, small cell lung cancer; UC, urothelial carcinoma.

Representative Singleplex and Multiplex IHC images are shown in Supplementary Fig. S1. Evaluable paired biopsy samples for Part 1 of the study were available for 9 patients in Group A (PR, *n* = 1; SD, *n* = 3; PD, *n* = 4; not evaluable, *n* = 1) and 11 patients in Group B (CR, *n* = 1; PR, *n* = 1; SD, *n* = 5; PD, *n* = 4). Itacitinib plus pembrolizumab treatment [Group A, A-1 (PD-[L]1 treatment-experienced) and A-2 (PD-[L]1 treatment-naïve)] had inconsistent effects on cell infiltration into the TME, regardless of treatment response or prior PD-(L)1 therapy (Fig. 2A). In contrast, parsaclisib plus pembrolizumab treatment (Group B, B-1/B-2) resulted in a significant decrease in FoxP3^+^-cell infiltration (*P* < 0.05; Fig. 2A), and a significant increase in CD8^+^:FoxP3^+^ ratio in nine of 11 biopsy pairs (*P* < 0.05; Fig. 2B). Notably, the significant increase in CD8^+^:FoxP3^+^ ratio was not accompanied by an increase in CD8^+^-cell infiltration in the TME (Fig. 2A), and there was no consistent association with clinical response (one CR, four SD, and four PD). In the 2 patients with decrease in CD8^+^:FoxP3^+^ ratio, the clinical response was SD and PD.

Because doses of parsaclisib examined in Part 1/Group B-1/B-2 (20–30 mg once daily) may also inhibit Teff function, in Part 2, a lower dose of parsaclisib (0.3 mg once daily) was evaluated in combination with pembrolizumab in PD-(L)1 treatment-naïve patients with SCLC, NSCLC, or UC. Evaluable paired biopsy samples were available for 9 patients in Part 2 (PR, *n* = 1; SD, *n* = 2; PD, *n* = 4; not evaluable, *n* = 2). The low-dose parsaclisib plus pembrolizumab treatment combination did not consistently modulate infiltration of cells examined, including CD8^+^ T cells, in either tumor or stroma (Fig. 2C). Although the tumor CD8^+^:FoxP3^+^ ratio increased in six of nine pairs of biopsies, the difference was not statistically significant (Fig. 2D).

### Plasma Proteomics

**FIGURE 3:**
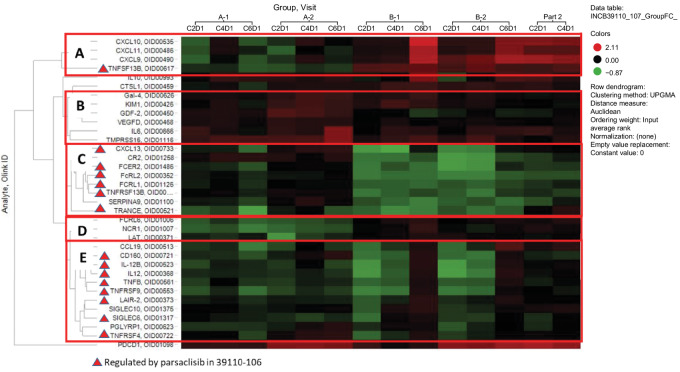
Heat map of plasma proteins significantly changed (log_2_ fold change) between cycle 2 day 1 and cycle 4 or 6 day 1 in treatment groups itacitinib + pembrolizumab (Part 1b Group A-1/A-2) or parsaclisib + pembrolizumab (Part 1b Group B-1/B-2, and Part 2). **A,** Interferon-inducible proteins reproducibly observed to increase following treatment with an anti-PD-1 antibody (e.g., pembrolizumab); IFN-inducible proteins were not induced in the itacitinib (JAK1 inhibitor) cotreatment arms (Group A-1/A-2). **B,** Proteins weakly increased in itacitinib-treated arm, no identifiable common pathway. **C,** Proteins decreased in the parsaclisib-treated (PI3Kδ inhibitor) arms, especially with high-dose parsaclisib (Group B-1/B-2), but less pronounced with low-dose (0.3 mg) parsaclisib (Part 2). **D,** Proteins decreased in the itacitinib-treated arms (Group A-1/A-2), involved in NK- and/or T-cell activation. **E,** Proteins decreased in high-dose parsaclisib-treated arms (Group B-1/B-2), highly enriched for proteins involved in T-cell, NK-cell, and macrophage function. C, cycle; D, day; JAK1, Janus kinase 1; NK, natural killer.

Plasma proteomic heat map analyses (Fig. 3) suggest that itacitinib cotreatment with pembrolizumab blocked activation of the interferon pathway, which is required for T-cell activation, and other proteins involved in natural killer (NK) and/or T-cell activation and function [Fc receptor-like 6 (FCRL6), NK cytotoxicity triggering receptor (NCR1), and linker for T-cell activation (LAT)]. Results also indicate that parsaclisib cotreatment with pembrolizumab potently inhibits proteins associated with macrophage and immune-cell function. Notable affected proteins included important cofactors for T-cell activation.

Nine unique proteins were significantly changed between cycle 1 and 2 in the itacitinib plus pembrolizumab combination groups (A-1/A-2; Supplementary Table S10). The most highly upregulated protein in Group A-2 [PD-(L)1 therapy-naïve] was PDCD1 (PD-1), which was likely attributable to soluble, stabilized PD-1–pembrolizumab complexes. The most downregulated proteins were NCR1 and FCRL6 in Group A-1 [PD-(L)]1 therapy-experienced, and LAT in Group A-2 [PD-(L)1 therapy-naïve], all of which are involved in NK and/or T-cell activation and function.

Twenty-seven unique proteins were differentially expressed between cycle 1 and 2 across the parsaclisib plus pembrolizumab combination groups (B-1/B-2, Part 2; Supplementary Table S10). PD-1 was the most upregulated protein in Groups B-2 and Part 2 [PD-(L)1 therapy-naïve]. The most downregulated proteins in the high-dose parsaclisib cotreatment Group B-1 [PD-(L)1 therapy-experienced] were chemokine C-X-C-motif-ligand (CXCL)13, IL12 and IL12 subunit beta (IL12B), and in Group B-2 [PD-(L)1 therapy-naïve] were complement receptor type 2 and IL12.

## Discussion

This phase Ib platform study evaluated the safety, efficacy, and pharmacodynamics of itacitinib (JAK1 inhibitor) or parsaclisib (PI3Kδ inhibitor) in combination with pembrolizumab (anti-PD-1) in patients with advanced or metastatic solid tumors. The study primary objective was to define a preliminary safety profile of itacitinib or parsaclisib in combination with pembrolizumab, and results were consistent with previously reported data (20, 23, 24). The MTD/PAD identified in the dose escalation Part 1a were 300 mg once daily for itacitinib, and 20 or 30 mg once daily for parsaclisib; these doses were administered to patients in the safety-expansion Part 1b. Discontinuations of parsaclisib due to AEs in Part 1a Group B (44.5%) were higher than that observed with monotherapy in relapsed or refractory B-cell lymphomas (19%; ref. 20). This observed difference could be due to the different tumor types and patient populations or the use of combination therapy with a CPI in the current study. Evolving pharmacokinetic and pharmacodynamic data for parsaclisib became available during the conduct of our study and suggested a lower dose may effectively modulate the TME, with preferential suppression of Treg function over Teff proliferation; therefore, a lower dose of parsaclisib at 0.3 mg once daily was selected for evaluation in Part 2. A parsaclisib dose of 0.3 mg was believed sufficient to reach the IC_90_ for Tregs, while remaining below the IC_50_ for Teffs (Incyte Corporation, data on file). In addition, PJP prophylaxis was required at higher doses of parsaclisib (≥1 mg once daily) in Part 1; this was not necessary with the lower parsaclisib dose selected for Part 2.

The study secondary objective was to investigate clinical efficacy and correlative pharmacodynamics. In Part 1a/Group B (parsaclisib 0.3–30 mg once daily or 2.5 mg every other day plus pembrolizumab 200 mg every 3 weeks), one patient achieved a CR (NSCLC) and 6 patients had a PR (adenocarcinoma, endometrial adenocarcinoma, NSCLC, cholangiocarcinoma, *n* = 1 each; SCLC, *n* = 2) as best response (ORR; 20.6%). This initial efficacy signal was not confirmed in Part 2 with parsaclisib 0.3 mg once daily plus pembrolizumab (PR achieved in 3 and 2 patients with SCLC and NSCLC, respectively; no objective responses in patients with UC). Consequently, the continued development of parsaclisib plus pembrolizumab was not supported. Tumor types investigated in Part 2 were selected in real-time based on results from Part 1b, but upon final data analysis, it appears results from Part 1 may not have fully supported the selection of SCLC, NSCLC, and UC for further evaluation in Part 2. Of note, treatment with parsaclisib monotherapy achieved an ORR of 25.5% in patients with relapsed or refractory diffuse large B-cell lymphoma (DLBCL), a subtype of non–Hodgkin lymphoma, which did not support further investigation of parsaclisib monotherapy in DLBCL (24).

Analysis of tumor biopsies demonstrated that itacitinib in combination with pembrolizumab had no consistent effect on immune-cell infiltration into the tumor or stroma. The combination of itacitinib plus pembrolizumab resulted in only minor changes in a small number (nine) of plasma proteins. Notably, IFN-inducible proteins, such as CXCL9 and CXCL10, which are hallmark markers of anti-PD-(L)1 treatment, were not induced in patients treated with itacitinib, suggesting that coadministration of a JAK1 inhibitor with pembrolizumab blocks activation of the IFN pathway, which is required for T-cell activation. Moreover, in Part 1b/Groups A-1/A-2, 4 patients achieved a PR with no remarkable efficacy observed in any one tumor type treated with itacitinib plus pembrolizumab. In a database analysis study of lung adenocarcinoma transcriptome profiles, JAK1 expression was positively correlated with immune-cell infiltration, suggesting a potential role of JAK1 in the immune response (25). In a noncancer but immune-related setting, JAK1 signaling has been implicated in the immune response in acute GVHD, and treatment with itacitinib demonstrated clinical benefit in a phase I study (23). Thus, although our study failed to show benefit of itacitinib plus pembrolizumab in solid tumors, further clinical investigation is required to better understand the role of JAK1 inhibition as a therapeutic strategy in this setting (26).

Pharmacodynamic evaluation of samples collected in Part 1 (Group B, B-1/B-2) demonstrated an increase in CD8^+^:FoxP3^+^ ratio with high-dose parsaclisib plus pembrolizumab; this change was not accompanied by an increase in CD8^+^-cell infiltration or clinical response, suggesting that a decrease in Tregs alone may not enhance the histopathologic manifestations of immune response in TME. Similarly, in Part 2, IHC analysis of samples collected during treatment with low-dose parsaclisib plus pembrolizumab demonstrated no reproducible effect on CD8^+^-cell infiltration, and although there was a trend toward increased CD8^+^:FoxP3^+^ ratio, the difference was not statistically significant. With high-dose and low-dose parsaclisib treatment used in study Part 1 and 2, respectively, translational results were not able to identify a parsaclisib dose that selectively inhibited PI3Kδ activity in Tregs versus Teffs. An increase in CD8^+^:FoxP3^+^ ratio without increased CD8^+^-cell infiltration appeared inadequate to enhance immune response in TME, and was not associated with clinical response. Observed changes in plasma proteins following parsaclisib plus pembrolizumab treatment are very similar to those reported with itacitinib plus parsaclisib combination in the INCB39110-106 study (NCT02559492; ref. 27). In contrast to the itacitinib arm, IFN-induced proteins CXCL9/10 were induced in response to parsaclisib plus pembrolizumab. Although CXCL9/10 induction was more prominent in the parsaclisib 0.3 mg once daily cohort, the data suggest that activation of T cells in the presence of PI3Kδ inhibition was at least partially intact. The majority of proteins downregulated by parsaclisib are involved in the regulation of immune cell activity, indicating that this treatment combination likely suppresses at least some aspects of immune activation. Studies with idelalisib (a PI3Kδ-selective inhibitor) have identified a difference in sensitivity of Treg versus Teff to PI3Kδ inhibition of cell proliferation that is one order of magnitude greater (28). This may partially explain the lack of an effect observed with parsaclisib in our study, with higher doses needed to modulate Teff activity in the TME.

Although a T-cell inflamed preclinical model demonstrated PI3Kδ inhibition together with PD-L1 blockade resulted in enhanced antitumor efficacy (18), studies with the MC38-OVA (murine colon adenocarcinoma) tumor model suggested that PI3Kδ inactivation (kinase-dead PI3Kδ^D910A^) negated the antitumor effects achieved with anti-PD-L1 (or anti-CTLA-4) in wild-type mice (29). In an LLC-OVA (Lewis lung carcinoma) tumor model, although tumors were unresponsive to anti-PD-L1 treatment in either wild-type or PI3Kδ^D910A^ mice, tumors were reduced by anti-CTLA-4 treatment in wild-type but not PI3Kδ^D910A^ mice (29). Therefore, antitumor effect of combined PI3Kδ inhibition and immune checkpoint blockade may depend on a number of factors, including tumor type and immune checkpoint receptor targeted (30).

In conclusion, PD-1 blockade in combination with either JAK1 or PI3Kδ inhibition in our study did not demonstrate significant clinical efficacy beyond that anticipated with pembrolizumab alone, despite encouraging preclinical activity in mouse models (15, 18). Although combination of parsaclisib and pembrolizumab showed modest clinical activity in CPI-pretreated patients, there were dosing limitations due to AEs and translational results were not able to identify a dose of parsaclisib that selectively modified Treg versus Teff biology to provide optimal PI3Kδ pathway modulation in the solid tumor types investigated. The overall clinical activity observed in this study does not support continued development of either combination in the selected solid tumor cohorts and dose regimens.

## Supplementary Material

Supplementary Figure 1 Supplementary Figure 1. Singleplex and Multiplex immunohistochemistry examples.

Supplementary Table 1 Supplementary Table 1. Summary of patient disposition (Part 1a Group A) (Full Analysis Set)

Supplementary Table 2 Supplementary Table 2. Summary of patient disposition (Part 1a Group B) (Full Analysis Set)

Supplementary Table 3 Supplementary Table 3. Summary of patient disposition (Part 1b Expansion Group A-1/A-2) (Full Analysis Set)

Supplementary Table 4 Summary of patient disposition (Part 1b Expansion Group B-1/B-2) (Full Analysis Set).

Supplementary Table 5 Summary of patient disposition (Part 2) (Full Analysis Set)

Supplementary Table 6 Summary of TEAEs by MedDRA preferred term [at least two patients (Part 1a Group A) or ≥10% of patients (Part 1a Group B, Part 1b, Part 2) in the safety population]

Supplementary Table 7 Supplementary Table 7. Serious TEAE ≥5% of patients by MedDRA preferred term (safety population).

Supplementary Table 8 Supplementary Table 8. Summary of steady state itacitinib pharmacokinetic parameters (cycle 2 day 1).

Supplementary Table 9 Supplementary Table 9. Summary of steady state parsaclisib pharmacokinetic parameters (cycle 2 day 1).

Supplementary Table 10 Supplementary Table 10. Plasma proteins differentially expressed between cycle 1 day 1 and cycle 2 day 1 with (A) itacitinib plus pembrolizumab (Part 1b Group A-1), or (B) parsaclisib plus pembrolizumab treatment (Part 1b Group B-1/B-2, and Part 2).
