# Correction: Combination of Itacitinib or Parsaclisib with Pembrolizumab in Patients with Advanced Solid Tumors: A Phase I Study

**DOI:** 10.1158/2767-9764.CRC-24-0133

**Published:** 2024-03-13

**Authors:** Pamela Munster, Nicholas Iannotti, Daniel C. Cho, John M. Kirkwood, Liza C. Villaruz, Geoffrey T. Gibney, F. Stephen Hodi, Niharika B. Mettu, Mark Jones, Jill Bowman, Michael Smith, Mani Lakshminarayanan, Steven O'Day

In the original version of this article (1), There is a typographical error in the “Total” column of the “Objective response, *n* (%)^g^” row in Table 3. The error has been corrected in the latest online HTML and PDF versions of the article. The editors regret this error.
