# Supplementary material for: Combination of Itacitinib or Parsaclisib with Pembrolizumab in Patients with Advanced Solid Tumors: A Phase I Study
Source: Cancer Res Commun. 2023 Dec 19;3(12):2572–84. doi: 10.1158/2767-9764.CRC-22-0461 (PMC10729644; doi:10.1158/2767-9764.CRC-22-0461)

Supplementary Figure 1. Singleplex and Multiplex immunohistochemistry examples. (**A**) Single-color Singleplex chromogenic assay in a patient with NSCLC (at screening compared with on-treatment), and (**B**) Five-color Multiplex immunohistochemistry assay in a patient with NSCLC (representative image of a single biopsy): orange, panCK (segmentation); blue, DAPI; red, CD3; green, CD8 (effector T cells); magenta, FoxP3 (regulatory T cells).

Abbreviations: DAPI, 4′,6-diamidino-2-phenylindole; FoxP3, forkhead box protein 3; NSCLC, non-small cell lung cancer; panCK, pan-cytokeratin.

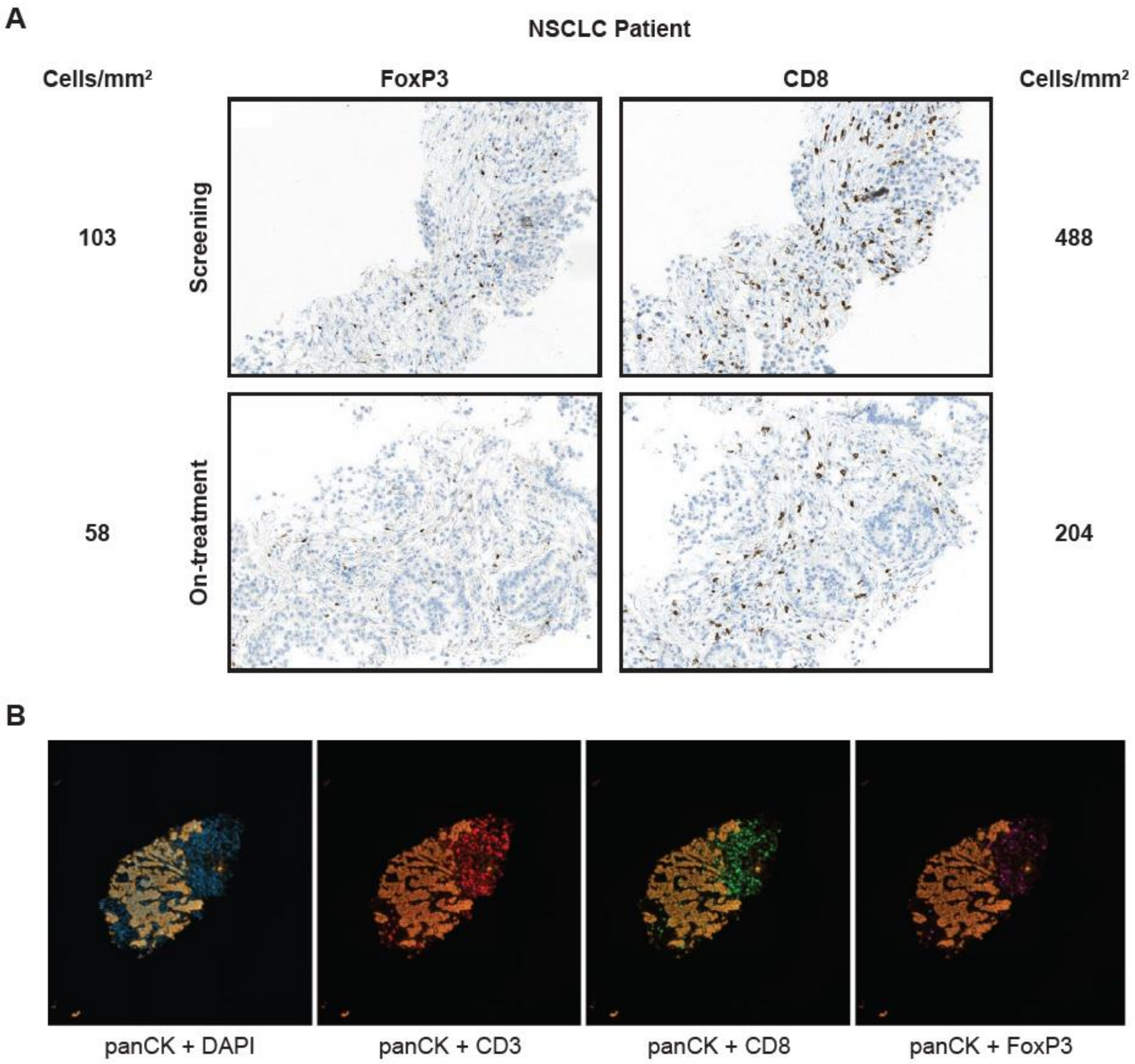

Supplement: Supplementary Figure 1 — Singleplex and Multiplex immunohistochemistry examples. [file crc-22-0461-s01.pdf]
