# Supplementary material for: Combination of Itacitinib or Parsaclisib with Pembrolizumab in Patients with Advanced Solid Tumors: A Phase I Study
Source: Cancer Res Commun. 2023 Dec 19;3(12):2572–84. doi: 10.1158/2767-9764.CRC-22-0461 (PMC10729644; doi:10.1158/2767-9764.CRC-22-0461)
Supplement: Supplementary Table 1 — Summary of patient disposition (Part 1a Group A) (Full Analysis Set) [file crc-22-0461-s02.pdf]

Supplementary Table 1. Summary of patient disposition (Part 1a Group A) (Full Analysis Set).

	**Dose level Itacitinib + Pembrolizumab**		
**Variable**	**300 mg QD/200 mg Q3W (*N*=4)**	**400 mg QD/200 mg Q3W (*N*=4)**	**Total (*N*=8)**
Number (%) of patients enrolled in the study	4 (100.0)	4 (100.0)	8 (100.0)
Number (%) of treated patients	4 (100.0)	4 (100.0)	8 (100.0)
Number (%) of patients with treatment ongoing	0	0	0
Number (%) of patients who completed treatment	0	0	0
**Number (%) of patients discontinued from treatment**	4 (100.0)	4 (100.0)	8 (100.0)
Primary reason of treatment discontinuation			
Adverse event	0	1 (25.0)	1 (12.5)
Progressive disease	3 (75.0)	3 (75.0)	6 (75.0)
Physician decision	1 (25.0)	0 (0.0)	1 (12.5)

Abbreviations: Q3W, every 3 weeks; QD, once daily.
