# Supplementary material for: Combination of Itacitinib or Parsaclisib with Pembrolizumab in Patients with Advanced Solid Tumors: A Phase I Study
Source: Cancer Res Commun. 2023 Dec 19;3(12):2572–84. doi: 10.1158/2767-9764.CRC-22-0461 (PMC10729644; doi:10.1158/2767-9764.CRC-22-0461)
Supplement: Supplementary Table 2 — Summary of patient disposition (Part 1a Group B) (Full Analysis Set) [file crc-22-0461-s03.pdf]

Supplementary Table 2. Summary of patient disposition (Part 1a Group B) (Full Analysis Set).

	**Dose level Parsaclisib + Pembrolizumab**							
**Variable**	**0.3 mg QD/ 200 mg Q3W (*N*=4)**	**1 mg QD/ 200 mg Q3W (*N*=4)**	**2.5 mg QOD/ 200 mg Q3W (*N*=9)**	**10 mg QD/ 200 mg Q3W (*N*=5)**	**15 mg QD/ 200 mg Q3W (*N*=5)**	**20 mg QD/ 200 mg Q3W (*N*=3)**	**30 mg QD/ 200 mg Q3W (*N*=4)**	**Total (*N*=34)**
Number (%) of patients enrolled in the study	4 (100.0)	4 (100.0)	9 (100.0)	5 (100.0)	5 (100.0)	3 (100.0)	4 (100.0)	34 (100.0)
Number (%) of treated patients	4 (100.0)	4 (100.0)	9 (100.0)	5 (100.0)	5 (100.0)	3 (100.0)	4 (100.0)	34 (100.0)
Number (%) of patients with treatment ongoing	0	0	0	0	0	0	0	0
Number (%) of patients who completed treatment	0	0	2 (22.2)	0	0	0	0	2 (5.9)
**Number (%) of patients discontinued from treatment**	4 (100.0)	4 (100.0)	7 (77.8)	5 (100.0)	5 (100.0)	3 (100.0)	4 (100.0)	32 (94.1)
Primary reason of treatment discontinuation								
Adverse event	3 (75.0)	0	2 (22.2)	3 (60.0)	2 (40.0)	2 (66.7)	2 (50.0)	14 (41.2)
Progressive disease	1 (25.0)	2 (50.0)	5 (55.6)	2 (40.0)	3 (60.0)	1 (33.3)	2 (50.0)	16 (47.1)
Death	0	1 (25.0)	0	0	0	0	0	1 (2.9)
Withdrawal by patient	0	1 (25.0)	0	0	0	0	0	1 (2.9)

Abbreviations: Q3W, every 3 weeks; QD, once daily; QOD, every other day.
