# Supplementary material for: Combination of Itacitinib or Parsaclisib with Pembrolizumab in Patients with Advanced Solid Tumors: A Phase I Study
Source: Cancer Res Commun. 2023 Dec 19;3(12):2572–84. doi: 10.1158/2767-9764.CRC-22-0461 (PMC10729644; doi:10.1158/2767-9764.CRC-22-0461)
Supplement: Supplementary Table 3 — Summary of patient disposition (Part 1b Expansion Group A-1/A-2) (Full Analysis Set) [file crc-22-0461-s04.pdf]

Supplementary Table 3. Summary of patient disposition (Part 1b Expansion Group A-1/A-2) (Full Analysis Set).

	**Itacitinib + Pembrolizumab**		
**Variable**	**Group A-1<br>300 mg QD/200 mg Q3W<br>(*N*=20)**	**Group A-2<br>300 mg QD/200 mg Q3W<br>(*N*=21)**	**Total<br>(*N* = 41)**
Number (%) of patients enrolled in the study	20 (100.0)	21 (100.0)	41 (100.0)
Number (%) of treated patients	20 (100.0)	21 (100.0)	41 (100.0)
Number (%) of patients with treatment ongoing	0	0	0
Number (%) of patients who completed treatment	0	0	0
**Number (%) of patients discontinued from treatment**	20 (100.0)	21 (100.0)	41 (100.0)
Primary reason of treatment discontinuation			
Adverse event	2 (10.0)	8 (38.1)	10 (24.4)
Progressive disease	17 (85.0)	12 (57.1)	29 (70.7)
Withdrawal by patient	1 (5.0)	1 (4.8)	2 (4.9)

Abbreviations: Q3W, every 3 weeks; QD, once daily.
