# Supplementary material for: Combination of Itacitinib or Parsaclisib with Pembrolizumab in Patients with Advanced Solid Tumors: A Phase I Study
Source: Cancer Res Commun. 2023 Dec 19;3(12):2572–84. doi: 10.1158/2767-9764.CRC-22-0461 (PMC10729644; doi:10.1158/2767-9764.CRC-22-0461)
Supplement: Supplementary Table 4 — Summary of patient disposition (Part 1b Expansion Group B-1/B-2) (Full Analysis Set). [file crc-22-0461-s05.pdf]

Supplementary Table 4. Summary of patient disposition (Part 1b Expansion Group B-1/B-2) (Full Analysis Set).

	Parsaclisib + Pembrolizumab				
**Variable**	**Group B-1 20 mg QD/ 200 mg Q3W (*N*=18)**	**Group B-2 20 mg QD/ 200 mg Q3W (*N*=12)**	**Group B-1 30 mg QD/ 200 mg Q3W (*N*=7)**	**Group B-2 30 mg QD/ 200 mg Q3W (*N*=12)**	**Total (*N*=49)**
Number (%) of patients enrolled in the study	18 (100.0)	12 (100.0)	7 (100.0)	12 (100.0)	49 (100.0)
Number (%) of treated patients	18 (100.0)	12 (100.0)	7 (100.0)	12 (100.0)	49 (100.0)
Number (%) of patients with treatment ongoing	0	0	0	0	0
Number (%) of patients who completed treatment	0	3 (25.0)	1 (14.3)	3 (25.0)	7 (14.3)
**Number (%) of patients discontinued from treatment**	18 (100.0)	9 (75.0)	6 (85.7)	9 (75.0)	42 (85.7)
Primary reason of treatment discontinuation					
Adverse event	3 (16.7)	2 (16.7)	1 (14.3)	0 (0.0)	6 (12.2)
Progressive disease	13 (72.2)	6 (50.0)	4 (57.1)	7 (58.3)	30 (61.2)
Death	0	0	0	1 (8.3)	1 (2.0)
Physician decision	2 (11.1)	0	0	0	2 (4.1)
Withdrawal by patient	0	1 (8.3)	1 (14.3)	0	2 (4.1)
Other	0	0	0	1 (8.3)	1 (2.0)

Number (%) of retreated patients	0	0	1 (14.3)	0	1 (2.0)
Number (%) of patients with retreatment ongoing	0	0	0	0	0
**Number (%) of patients discontinued from retreatment**	0	0	1 (14.3)	0	1 (2.0)
Primary reason of retreatment discontinuation					
Adverse event	0	0	1 (14.3)	0	1 (2.0)

Abbreviations: Q3W, every 3 weeks; QD, once daily.
