# Supplementary material for: Combination of Itacitinib or Parsaclisib with Pembrolizumab in Patients with Advanced Solid Tumors: A Phase I Study
Source: Cancer Res Commun. 2023 Dec 19;3(12):2572–84. doi: 10.1158/2767-9764.CRC-22-0461 (PMC10729644; doi:10.1158/2767-9764.CRC-22-0461)
Supplement: Supplementary Table 5 — Summary of patient disposition (Part 2) (Full Analysis Set) [file crc-22-0461-s06.pdf]

Supplementary Table 5. Summary of patient disposition (Part 2) (Full Analysis Set).

	Parsaclisib + Pembrolizumab			
**Variable**	**SCLC[a] 0.3 mg QD/ 200 mg Q3W (*N*=14)**	**NSCLC 0.3 mg QD/ 200 mg Q3W (*N*=8)**	**UC 0.3 mg QD/ 200 mg Q3W (*N*=5)**	**Total (*N*=27)**
Number (%) of patients enrolled in the study	14 (100.0)	8 (100.0)	5 (100.0)	27 (100.0)
Number (%) of treated patients	14 (100.0)	8 (100.0)	5 (100.0)	27 (100.0)
Number (%) of patients with treatment ongoing	0	0	0	0
Number (%) of patients who completed treatment	0	1 (12.5)	0	1 (3.7)
**Number (%) of patients discontinued from treatment**	14 (100.0)	7 (87.5)	5 (100.0)	26 (96.3)
Primary reason of treatment discontinuation				
Adverse event	7 (50.0)	1 (12.5)	1 (20.0)	9 (33.3)
Progressive disease	6 (42.9)	4 (50.0)	3 (60.0)	13 (48.1)
Physician decision	0	1 (12.5)	0	1 (3.7)
Withdrawal by patient	1 (7.1)	1 (12.5)	1 (20.0)	3 (11.1)

Abbreviations: NSCLC, non-small cell lung cancer; Q3W, every 3 weeks; QD, once daily; SCLC, small cell lung cancer; UC, urothelial carcinoma.
[a]Included one patient who received parsaclisib at a starting dose of 20 mg QD.
