# Supplementary material for: Combination of Itacitinib or Parsaclisib with Pembrolizumab in Patients with Advanced Solid Tumors: A Phase I Study
Source: Cancer Res Commun. 2023 Dec 19;3(12):2572–84. doi: 10.1158/2767-9764.CRC-22-0461 (PMC10729644; doi:10.1158/2767-9764.CRC-22-0461)
Supplement: Supplementary Table 6 — Summary of TEAEs by MedDRA preferred term [at least two patients (Part 1a Group A) or ≥10% of patients (Part 1a Group B, Part 1b, Part 2) in the safety population] [file crc-22-0461-s07.pdf]

Supplementary Table 6. Summary of TEAEs by MedDRA preferred term [at least two patients (Part 1a Group A) or ≥10% of patients (Part 1a Group B, Part 1b, Part 2) in the safety population].

	**Part 1 Itacitinib + Pembrolizumab or Parsaclisib + Pembrolizumab**				**Part 2 Parsaclisib + Pembrolizumab**			
**Preferred term, *n* (%)**	**Part 1a Group A (itacitinib) (*N*=8)**	**Part 1a Group B (parsaclisib) (*N*=34)**	**Part 1b Group A-1/A-2 (itacitinib) (*N*=41)**	**Part 1b Group B-1/B-2 (parsaclisib) (*N*=49)**	**SCLC[a] 0.3 mg QD/ 200 mg Q3W (*N*=14)**	**NSCLC 0.3 mg QD/ 200 mg Q3W (*N*=8)**	**UC 0.3 mg QD/ 200 mg Q3W (*N*=5)**	**Part 2 Total (*N*=27)**
Any TEAE	8 (100.0)	34 (100.0)	41 (100.0)	49 (100.0)	14 (100.0)	8 (100.0)	5 (100.0)	27 (100.0)
Anemia	5 (62.5)	6 (17.6)	13 (31.7)	14 (28.6)	4 (28.6)	0	3 (60.0)	7 (25.9)
Nausea	5 (62.5)	14 (41.2)	19 (46.3)	20 (40.8)	6 (42.9)	3 (37.5)	1 (20.0)	10 (37.0)
Fatigue	4 (50.0)	19 (55.9)	18 (43.9)	21 (42.9)	7 (50.0)	1 (12.5)	2 (40.0)	10 (37.0)
Decreased appetite	4 (50.0)	9 (26.5)	10 (24.4)	12 (24.5)	5 (35.7)	2 (25.0)	2 (40.0)	9 (33.3)
Hypokalemia	3 (37.5)	6 (17.6)	3 (7.3)	7 (14.3)	1 (7.1)	2 (25.0)	1 (20.0)	4 (14.8)
Pyrexia	3 (37.5)	10 (29.4)	13 (31.7)	16 (32.7)	3 (21.4)	1 (12.5)	0	4 (14.8)
Weight decreased	3 (37.5)	6 (17.6)	1 (2.4)	8 (16.3)	5 (35.7)	1 (12.5)	1 (20.0)	7 (25.9)
Back pain	2 (25.0)	7 (20.6)	4 (9.8)	12 (24.5)	5 (35.7)	4 (50.0)	1 (20.0)	10 (37.0)
Depression	2 (25.0)	3 (8.8)	0	3 (6.1)	0	1 (12.5)	0	1 (3.7)
Dizziness	2 (25.0)	7 (20.6)	4 (9.8)	3 (6.1)	3 (21.4)	1 (12.5)	0	4 (14.8)
Dyspnea	2 (25.0)	4 (11.8)	9 (22.0)	6 (12.2)	2 (14.3)	3 (37.5)	2 (40.0)	7 (25.9)
Fall	2 (25.0)	3 (8.8)	1 (2.4)	4 (8.2)	4 (28.6)	1 (12.5)	0	5 (18.5)

Hypomagnesemia	2 (25.0)	1 (2.9)	1 (2.4)	0	2 (14.3)	1 (12.5)	0	3 (11.1)
Pain in extremity	2 (25.0)	0	2 (4.9)	5 (10.2)	0	0	1 (20.0)	1 (3.7)
Pleural effusion	2 (25.0)	2 (5.9)	5 (12.2)	5 (10.2)	1 (7.1)	1 (12.5)	1 (20.0)	3 (11.1)
Urinary tract infection	2 (25.0)	8 (23.5)	4 (9.8)	6 (12.2)	4 (28.6)	2 (25.0)	1 (20.0)	7 (25.9)
Vomiting	2 (25.0)	11 (32.4)	11 (26.8)	12 (24.5)	3 (21.4)	1 (12.5)	0	4 (14.8)
Aspartate aminotransferase increased	0	7 (20.6)	4 (9.8)	5 (10.2)	0	0	0	0
Diarrhea	1 (12.5)	15 (44.1)	11 (26.8)	15 (30.6)	4 (28.6)	3 (37.5)	1 (20.0)	8 (29.6)
Constipation	0	10 (29.4)	12 (29.3)	9 (18.4)	2 (14.3)	3 (37.5)	1 (20.0)	6 (22.2)
Pruritus	0	10 (29.4)	3 (7.3)	11 (22.4)	4 (28.6)	1 (12.5)	1 (20.0)	6 (22.2)
Abdominal pain	1 (12.5)	8 (23.5)	11 (26.8)	12 (24.5)	4 (28.6)	3 (37.5)	1 (20.0)	8 (29.6)
Cough	0	6 (17.6)	6 (14.6)	12 (24.5)	3 (21.4)	0	l (20.0)	4 (14.8)
Arthralgia	1 (12.5)	5 (14.7)	6 (14.6)	10 (20.4)	2 (14.3)	2 (25.0)	0	4 (14.8)
Dehydration	1 (12.5)	6 (17.6)	8 (19.5)	4 (8.2)	5 (35.7)	1 (12.5)	1 (20.0)	7 (25.9)
Dysphagia	0	1 (2.9)	3 (7.3)	0	4 (28.6)	0	2 (40.0)	6 (22.2)
Pneumonia	1 (12.5)	5 (14.7)	3 (7.3)	2 (4.1)	4 (28.6)	2 (25.0)	0	6 (22.2)
Upper respiratory tract infection	0	5 (14.7)	2 (4.9)	4 (8.2)	2 (14.3)	4 (50.0)	0	6 (22.2)
Hypotension	1 (12.5)	3 (8.8)	2 (4.9)	3 (6.1)	4 (28.6)	1 (12.5)	0	5 (18.5)
Insomnia	0	7 (20.6)	3 (7.3)	5 (10.2)	2 (14.3)	1 (12.5)	2 (40.0)	5 (18.5)

Anxiety	1 (12.5)	5 (14.7)	5 (12.2)	4 (8.2)	1 (7.1)	3 (37.5)	0	4 (14.8)
Headache	1 (12.5)	4 (11.8)	6 (14.6)	9 (18.4)	1 (7.1)	2 (25.0)	1 (20.0)	4 (14.8)
Hypothyroidism	0	3 (8.8)	0	1 (2.0)	2 (14.3)	1 (12.5)	1 (20.0)	4 (14.8)
Muscular weakness	0	3 (8.8)	2 (4.9)	4 (8.2)	3 (21.4)	1 (12.5)	0	4 (14.8)
Maculopapular rash	0	0	3 (7.3)	3 (6.1)	3 (21.4)	0	1 (20.0)	4 (14.8)
Acute respiratory failure	0	0	0	0	1 (7.1)	0	2 (40.0)	3 (11.1)
Hyponatremia	0	3 (8.8)	4 (9.8)	8 (16.3)	2 (14.3)	0	1 (20.0)	3 (11.1)
Musculoskeletal chest pain	0	2 (5.9)	2 (4.9)	1 (2.0)	2 (14.3)	1 (12.5)	0	3 (11.1)
Tachycardia	1 (12.5)	0	2 (4.9)	3 (6.1)	2 (14.3)	1 (12.5)	0	3 (11.1)
White blood cell count decreased	0	1 (2.9)	0	3 (6.1)	1 (7.1)	1 (12.5)	1 (20.0)	3 (11.1)
Chills	0	6 (17.6)	3 (7.3)	2 (4.1)	1 (7.1)	0	1 (20.0)	2 (7.4)
Confusional state	0	5 (14.7)	1 (2.4)	3 (6.1)	2 (14.3)	0	0	2 (7.4)
Gastroesophageal reflux disease	1 (12.5)	5 (14.7)	2 (4.9)	1 (2.0)	2 (14.3)	0	0	2 (7.4)
Abdominal pain upper	1 (12.5)	4 (11.8)	2 (4.9)	2 (4.1)	1 (7.1)	0	0	1 (3.7)
Alanine aminotransferase increased	0	4 (11.8)	5 (12.2)	4 (8.2)	0	0	0	0

Blood alkaline phosphatase increased	1 (12.5)	4 (11.8)	2 (4.9)	5 (10.2)	0	0	0	0
Dry skin	0	4 (11.8)	0	1 (2.0)	1 (7.1)	0	0	1 (3.7)
Hyperglycemia	0	4 (11.8)	2 (4.9)	7 (14.3)	0	1 (12.5)	0	1 (3.7)
Hypersensitivity	0	4 (11.8)	0	0	0	0	0	0
Stomatitis	0	4 (11.8)	0	5 (10.2)	1 (7.1)	1 (12.5)	0	2 (7.4)
Malignant neoplasm progression	0	3 (8.8)	8 (19.5)	3 (6.1)	1 (7.1)	0	0	1 (3.7)
Musculoskeletal pain	0	2 (5.9)	5 (12.2)	3 (6.1)	0	1 (12.5)	1 (20.0)	2 (7.4)
Edema peripheral	0	3 (8.8)	4 (9.8)	8 (16.3)	1 (7.1)	1 (12.5)	0	2 (7.4)
Neutrophil count decrease	0	1 (2.9)	0	6 (12.2)	1 (7.1)	1 (12.5)	0	2 (7.4)
Pollakiuria	0	0	1 (2.4)	6 (12.2)	0	0	0	0
Rash	0	3 (8.8)	3 (7.3)	6 (12.2)	0	0	1 (20.0)	1 (3.7)
Dysgeusia	1 (12.5)	2 (5.9)	2 (4.9)	5 (10.2)	0	0	0	0
Hypoalbuminemia	0	0	3 (7.3)	5 (10.2)	1 (7.1)	0	1 (20.0)	2 (7.4)
Hypoxia	1 (12.5)	0	0	5 (10.2)	0	0	0	0
Influenza-like illness	0	1 (2.9)	2 (4.9)	5 (10.2)	0	0	0	0

Abbreviations: MedDRA, Medical Dictionary for Regulatory Activities; Q3W, every 3 weeks; QD, once daily; TEAE, treatment-emergent adverse event.
[a]Included one patient who received parsaclisib at a starting dose of 20 mg QD.
