# Supplementary material for: Combination of Itacitinib or Parsaclisib with Pembrolizumab in Patients with Advanced Solid Tumors: A Phase I Study
Source: Cancer Res Commun. 2023 Dec 19;3(12):2572–84. doi: 10.1158/2767-9764.CRC-22-0461 (PMC10729644; doi:10.1158/2767-9764.CRC-22-0461)
Supplement: Supplementary Table 7 — Serious TEAE ≥5% of patients by MedDRA preferred term (safety population). [file crc-22-0461-s08.pdf]

Supplementary Table 7. Serious TEAE ≥5% of patients by MedDRA preferred term (safety population).

	Part 1 Itacitinib + Pembrolizumab or Parsaclisib + Pembrolizumab				Part 2 Parsaclisib + Pembrolizumab			
**Preferred term, *n* (%)**	**Part 1a Group A (itacitinib) (*N*=8)**	**Part 1a Group B (parsaclisib) (*N*=34)**	**Part 1b Group A-1/A-2 (itacitinib) (*N*=41)**	**Part 1b Group B-1/B-2 (parsaclisib) (*N*=49)**	**SCLC[a] 0.3 mg QD/ 200 mg Q3W (*N*=14)**	**NSCLC 0.3 mg QD/ 200 mg Q3W (*N*=8)**	**UC 0.3 mg QD/ 200 mg Q3W (*N*=5)**	**Part 2 Total (*N*=27)**
Any serious TEAE	6 (75.0)	23 (67.6)	20 (48.8)	27 (55.1)	8 (57.1)	5 (62.5)	3 (60.0)	16 (59.3)
Pyrexia	2 (25.0)	1 (2.9)	3 (7.3)	0	0	0	0	0
Back pain	2 (25.0)	0	0	0	0	0	0	0
Atrial fibrillation	1 (12.5)	0	0	3 (6.1)	0	0	0	0
Lower GI hemorrhage	1 (12.5)	1 (2.9)	0	0	0	0	0	0
Pneumonia	1 (12.5)	4 (11.8)	2 (4.9)	1 (2.0)	4 (28.6)	2 (25.0)	0	6 (22.2)
Lung cancer metastatic	1 (12.5)	1 (2.9)	0	0	0	0	0	0
Pleural effusion	1 (12.5)	0	1 (2.4)	1 (2.0)	1 (7.1)	0	1 (20.0)	2 (7.4)
Urinary tract infection	0	4 (11.8)	3 (7.3)	0	0	0	0	0
Small intestinal obstruction	0	2 (5.9)	0	1 (2.0)	0	0	0	0
Sepsis	0	2 (5.9)	1 (2.4)	3 (6.1)	1 (7.1)	0	0	1 (3.7)

Malignant neoplasm progression	0	2 (5.9)	8 (19.5)	3 (6.1)	1 (7.1)	0	0	1 (3.7)
Pneumonitis	0	2 (5.9)	0	0	0	0	0	0
Pulmonary embolism	0	1 (2.9)	3 (7.3)	0	0	0	0	0
Metastatic SCLC	0	0	0	0	2 (14.3)	0	0	2 (7.4)
Pneumothorax	0	1 (2.9)	0	0	0	2 (25.0)	0	2 (7.4)
Acute respiratory failure	0	0	0	0	0	0	2 (40.0)	2 (7.4)

Abbreviations: GI, gastrointestinal; MedDRA, Medical Dictionary for Regulatory Activities; NSCLC, non-small cell lung cancer; Q3W, every 3 weeks; QD, once daily; SCLC, small cell lung cancer; TEAE, treatment-emergent adverse event; UC, urothelial carcinoma.
[a]Included one patient who received parsaclisib at a starting dose of 20 mg QD.
