# Supplementary material for: Combination of Itacitinib or Parsaclisib with Pembrolizumab in Patients with Advanced Solid Tumors: A Phase I Study
Source: Cancer Res Commun. 2023 Dec 19;3(12):2572–84. doi: 10.1158/2767-9764.CRC-22-0461 (PMC10729644; doi:10.1158/2767-9764.CRC-22-0461)
Supplement: Supplementary Table 8 — Summary of steady state itacitinib pharmacokinetic parameters (cycle 2 day 1). [file crc-22-0461-s09.pdf]

Supplementary Table 8. Summary of steady state itacitinib pharmacokinetic parameters (cycle 2 day 1).

		Parameter[a]				
**Dose**	***N***	**AUC_{τ} (nM·h)**	**CL_{ss}/F (L/h)**	**C_{max} (nM)**	**C_{tau} (nM)[b]**	**T_{max} (h)**
300 mg QD	34	6,500 (5,110), 5,000	144 (129), 108	1,050 (625), 879	32.2 (41.8)	2.01 (0.833, 4.30)
400 mg QD	3[c]	3,820 (2,370), 3,180	287 (247), 227	681 (283), 641	21.9 (18.5)	2.45 (1.53, 2.47)

AUC_{τ}, area under the steady state concentration-time curve over a dosing interval; CL_{ss}/F, apparent oral dose clearance at steady state; C_{max}, maximum observed concentration; C_{tau}, concentration at the end of the dosing interval (i.e., predose); QD, once daily; SD, standard deviation; T_{max}, time to maximum concentration.

[a] Data presented as mean (SD), geometric mean, with exception T_{max} presented as median (minimum, maximum).

[b] Geometric mean not calculated because of zero values.

[c] $N = 4$ for C_{tau}.
