# Supplementary material for: Combination of Itacitinib or Parsaclisib with Pembrolizumab in Patients with Advanced Solid Tumors: A Phase I Study
Source: Cancer Res Commun. 2023 Dec 19;3(12):2572–84. doi: 10.1158/2767-9764.CRC-22-0461 (PMC10729644; doi:10.1158/2767-9764.CRC-22-0461)
Supplement: Supplementary Table 9 — Summary of steady state parsaclisib pharmacokinetic parameters (cycle 2 day 1). [file crc-22-0461-s10.pdf]

Supplementary Table 9. Summary of steady state parsaclisib pharmacokinetic parameters (cycle 2 day 1).

		Parameter[a]				
**Dose**	***N***	**AUC_τ (nM·h)**	**CL_{ss}/F (L/h)**	**C_{max} (nM)**	**C_{tau} (nM)**	**T_{max} (h)**
0.3 mg QD	25	295 (154), 266	2.84 (1.18), 2.60	26.9 (7.70), 25.8	4.76 (6.41)	1.0 (0.75, 2.5)
1 mg QD	4	1,010 (493), 916	2.76 (1.28), 2.52	105 (45.2), 98.4	13.6 (15.9)	1.0 (0.95, 2.1)
2.5 mg QOD	4	2,280 (441), 2,250	2.60 (0.469), 2.57	155 (45.8), 150	8.43 (6.83)	1.0 (0.87, 1.8)
10 mg QD	4	7,480 (3,530), 6,930	3.56 (1.38), 3.33	834 (280), 801	106 (67.3), 90.7	1.1 (1.0, 1.7)
15 mg QD	3	10,400 (2,840), 10,100	3.51 (0.955), 3.43	1,190 (293), 1,160	132 (50.3), 124	0.90 (0.87, 1.2)
20 mg QD	23[b]	17,000 (7,180), 15,600	3.21 (1.36), 2.96	1,840 (657), 1,690	265 (230)	1.0 (0.83, 5.0)
30 mg QD	15[c]	26,900 (12,200), 24,600	3.05 (1.23), 2.81	2,580 (723), 2,480	505 (436), 374	1.1 (0.93, 2.4)

Abbreviations: AUC_τ, area under the steady state concentration-time curve over a dosing interval; CL_{ss}/F, apparent oral dose clearance at steady state; C_{max}, maximum observed concentration; C_{tau}, concentration at the end of the dosing interval (i.e., predose); QD, once daily; QOD, every other day; SD, standard deviation; T_{max}, time to maximum concentration.

[a] Data presented as mean (SD), geometric mean, with exception T_{max} presented as median (minimum, maximum).

[b] *N* = 25 for C_{tau}.

[c] *N* = 16 for C_{tau}.
