# Supplementary material for: Combination of Itacitinib or Parsaclisib with Pembrolizumab in Patients with Advanced Solid Tumors: A Phase I Study
Source: Cancer Res Commun. 2023 Dec 19;3(12):2572–84. doi: 10.1158/2767-9764.CRC-22-0461 (PMC10729644; doi:10.1158/2767-9764.CRC-22-0461)
Supplement: Supplementary Table 10 — Plasma proteins differentially expressed between cycle 1 day 1 and cycle 2 day 1 with (A) itacitinib plus pembrolizumab (Part 1b Group A-1), or (B) parsaclisib plus pembrolizumab treatment (Part 1b Group B-1/B-2, and Part 2). [file crc-22-0461-s11.pdf]

Supplementary Table 10. Plasma proteins differentially expressed between cycle 1 day 1 and cycle 2 day 1 with (**A**) itacitinib plus pembrolizumab (Part 1b Group A-1), or (**B**) parsaclisib plus pembrolizumab treatment (Part 1b Group B-1/B-2, and Part 2).

Group	Analyte	Mean Log2 FC	FDR_p	Group	Analyte	Mean Log2 FC	FDR_p	Group	Analyte	Mean Log2 FC	FDR_p
**Itacitinib + pembrolizumab**											
A-1	NCR1	-0.474	0.01419	A-2	LAT	-0.758	0.03009				
A-1	FCRL6	-0.421	0.02294	A-2	Gal-4	0.419	0.04593				
A-1	TMPRSS15	0.505	0.02289	A-2	VEGFD	0.437	0.00208				
				A-2	KIM1	0.540	0.04970				
				A-2	GDF-2	0.818	0.01568				
				A-2	PDCD1	1.291	0.00004				
**Parsaclisib + pembrolizumab**											
B-1	CXCL13	-0.782	0.00777	B-2	CR2	-0.824	0.00265	Part 2	SERPINA9	-0.459	0.02454
B-1	IL12	-0.766	0.01568	B-2	IL12	-0.812	0.01419	Part 2	FcRL2	-0.413	0.00000
B-1	IL-12B	-0.746	0.01568	B-2	FCER2	-0.793	0.00004	Part 2	CXCL11	0.540	0.04949
B-1	TNFRSF9	-0.655	0.01619	B-2	IL-12B	-0.791	0.01419	Part 2	TNFSF13B	0.575	0.00769
B-1	FCER2	-0.611	0.02888	B-2	CXCL13	-0.703	0.01419	Part 2	IL10	0.614	0.02812
B-1	FcRL2	-0.608	0.00390	B-2	FcRL2	-0.655	0.00034	Part 2	IL6	0.633	0.04645
B-1	TNFRSF13B	-0.593	0.00720	B-2	TNFRSF9	-0.590	0.00034	Part 2	CXCL10	0.947	0.00790
B-1	CD160	-0.584	0.02721	B-2	CD160	-0.563	0.01419	Part 2	CXCL9	1.168	0.00231
B-1	CR2	-0.581	0.00601	B-2	CCL19	-0.553	0.02603	Part 2	PDCD1	1.514	0.00000
B-1	SIGLEC6	-0.553	0.01419	B-2	SERPINA9	-0.489	0.03388				
B-1	TNFRSF4	-0.507	0.01736	B-2	TNFRSF13B	-0.452	0.00142				
B-1	TNFB	-0.504	0.04529	B-2	FCRL1	-0.430	0.01419				
B-1	TRANCE	-0.488	0.02487	B-2	PGLYRP1	-0.413	0.04881				
B-1	FCRL1	-0.456	0.04593	B-2	CTSL1	0.412	0.04539				
B-1	SIGLEC10	-0.444	0.01419	B-2	TNFSF13B	0.706	0.01419				
B-1	LAIR-2	-0.443	0.04751	B-2	PDCD1	1.394	0.00004				
B-1	TNFSF13B	0.815	0.01419								

Abbreviations: FC, fold change; FDR, false discovery rate.
